# Robust network stability of mosquitoes and human pathogens of medical importance

**DOI:** 10.1186/s13071-022-05333-4

**Published:** 2022-06-20

**Authors:** Donald A. Yee, Catherine Dean Bermond, Limarie J. Reyes-Torres, Nicole S. Fijman, Nicole A. Scavo, Joseph Nelsen, Susan H. Yee

**Affiliations:** 1 School of Biological, Environmental, and Earth Sciences, University of Southern Mississippi, 118 College Drive, Hattiesburg, MS 39406 USA; 2 Gulf Ecosystem Measurement and Modeling Division, U.S. Environmental Protection Agency, 1 Sabine Island Drive, Gulf Breeze, FL 32561 USA

**Keywords:** Arbovirus, Culicidae, Extinction curves, Network analysis, Pathogen, Vector

## Abstract

**Background:**

The exact number of mosquito species relevant to human health is unknown, posing challenges in understanding the scope and breadth of vector–pathogen relationships, and how resilient mosquito vector–pathogen networks are to targeted eradication of vectors.

**Methods:**

We performed an extensive literature survey to determine the associations between mosquito species and their associated pathogens of human medical importance. For each vector–pathogen association, we then determined the strength of the associations (i.e., natural infection, lab infection, lab dissemination, lab transmission, known vector). A network analysis was used to identify relationships among all pathogens and vectors. Finally, we examined how elimination of either random or targeted species affected the extinction of pathogens.

**Results:**

We found that 88 of 3578 mosquito species (2.5%) are known vectors for 78 human disease-causing pathogens; however, an additional 243 species (6.8%) were identified as potential or likely vectors, bringing the total of all mosquitos implicated in human disease to 331 (9.3%). Network analysis revealed that known vectors and pathogens were compartmentalized, with the removal of six vectors being enough to break the network (i.e., cause a pathogen to have no vector). However, the presence of potential or likely vectors greatly increased redundancies in the network, requiring more than 41 vectors to be eliminated before breaking the network.

**Conclusion:**

Although < 10% of mosquitoes are involved in transmitting pathogens that cause human disease, our findings point to inherent robustness in global mosquito vector–pathogen networks.

**Graphical Abstract:**

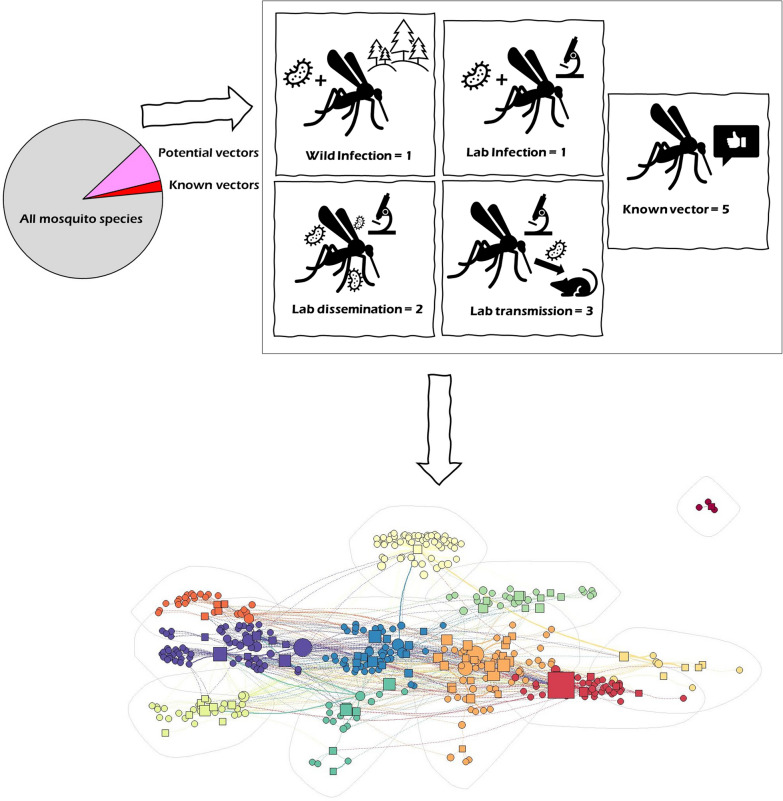

**Supplementary Information:**

The online version contains supplementary material available at 10.1186/s13071-022-05333-4.

## Background

Mosquitoes are one of the main animal agents of human infectious disease, and the pathogens they transmit have likely been a selective force for human evolution (e.g., sickle cell disease [1]) and have also had profound political and historical effects [2]. With few exceptions, humans across the globe are susceptible to a wide range of pathogens that are carried by adult mosquitoes, including debilitating and sometimes fatal diseases like malaria, dengue, yellow fever, and West Nile encephalitis. Considerable time and treasure have been spent on attempting to suppress mosquito populations, with the hope of controlling pathogen transmission among humans. Although there has been some success in these endeavors, even the best control approaches may be restricted to narrow geographical ranges, and often yield fleeting results (e.g., *Aedes aegypti* in Brazil [3]). At present we lack a comprehensive list of medically important mosquitoes, hampering our ability to target all species involved in pathogen transmission. Moreover, the relationships between mosquitoes and pathogens are varied, ranging from natural infection by a pathogen, to being the known causative agent of human pathogen transmission. As relationships between vectors and pathogens vary among species for the same pathogen, it is likely that even with the elimination of a focal species, other competent vectors exist.

To eliminate or reduce mosquito-borne disease burden, suppression of mosquito-borne pathogens often occurs for pairs of vectors and pathogens [4, 5]. However, this often ignores the fact that pathogens frequently have several competent known or suspected vectors, and individual vectors can be responsible for transmitting several pathogens. One useful approach to consolidating these complex relationships for human disease is via ecological network modeling [6, 7], which can simultaneously consider the relationships among pathogens and vector species (i.e., a cluster) and the relationships among these clusters. Such an approach can lead to novel understanding of the ecological, epidemiological, and evolutionary patterns among vertebrate hosts, vectors, and transmitted pathogens [6, 8, 9]. Unlike some beneficial interactions among species (e.g., plants-pollinators [10]), vector–pathogen networks are inherently asymmetrical, with the loss of a pathogen having a positive or neutral effect on vectors, and the loss of vectors having negative effects on pathogens.

Here we quantified the pathogens of human medical importance that are transmitted by mosquitoes, and then for each pathogen assessed the current state of knowledge for the relationships with potential or known mosquito species (i.e., wild infection, lab infection, lab dissemination, lab transmission, or known vector). Our approach to use all associations (both in the lab and from the wild) across the entire geographical range of vector–pathogen networks allows for a broad understanding of the difficulties and challenges of mosquito-borne disease elimination. This broad approach assumes that range expansion and species introductions are still likely to occur, and that our current knowledge regarding all species implicated in mosquito-borne disease is incomplete. This process also allowed us to quantify, for the first time, the number and species of mosquitoes of human medical importance. This data set was then used in a network analysis to ascertain the associations of vectors and pathogens within and among clusters. We use these data to consider both known associations among vectors and pathogens, and potential or likely vectors for each pathogen. Finally, we determined the degree to which random or targeted removal of mosquito species would break the network (i.e., lead to the extinction of one or more pathogens).

## Methods

We conducted an extensive review of the scientific literature, secondary sources (e.g., https://wwwn.cdc.gov/arbocat/), and authoritative books on mosquitoes or human disease e.g. [11–13] to establish a comprehensive list of pathogens that are known to be transmitted by mosquitoes and that cause documented human illness (e.g., fever, death). In one case, Fort Sherman virus, although this pathogen can cause human illness and has been identified as mosquito-borne, no data exist to specify which species are involved (although *Aedes* are implicated [14]). All four serotypes of dengue were consolidated to a single pathogen group, as were all species of *Plasmodium*. Consolidating these pathogens in this way was consistent with our global approach to understanding the vector–pathogen relationships.

Next, we identified all mosquito species that have a documented role in the scientific literature or secondary sources in carrying, disseminating, or transmitting each identified pathogen. Searches were conducted using scientific literature databases (e.g., Web of Science, Google Scholar) to search for each pathogen, with abstracts and documents reviewed to identify articles with relevant information (e.g., field or lab studies documenting infection, dissemination, or transmission; cited references) on mosquito species associated with that pathogen. Cited references within articles suggesting or identifying potential mosquito-related information were also reviewed, as well as secondary sources (e.g., https://wwwn.cdc.gov/arbocat/) and certain texts, e.g. [11, 12], until additional searches produced no new relationships that hadn’t already been identified. As a final check for completeness, we searched the databases Crossref (https://www.crossref.org) and Entrez (https://www.ncbi.nlm.nih.gov/search/) for every possible pairwise combination of identified pathogens and identified mosquito species, to verify that no potential relationship had been overlooked. Genus-level vector information (i.e., unknown species) was used for a pathogen only if it represented a new genus for which no species-level information was otherwise available. Genus-level information was retained for ten vectors, paired with six pathogens, and was assumed to be uniquely associated with each pathogen (e.g., *Mansonia* sp. paired with Guama virus not assumed to be the same species as *Mansonia* sp. paired with western equine encephalitis). In all cases we assumed species names were used in the sensu stricto (s.s.) sense (e.g., *Anopheles gambiae*) based on the publications that listed them. We could not discern this in all cases, as many papers did not list species as s.s. or sensu lato (s.l.), but given the nature of those publications, we assumed they were s.s. Herein we also hold to traditional taxonomic affiliations for the genus *Aedes* given the current uncertainty of new designations. Finally, one observation of wild infection of St. Louis encephalitis virus in *Toxorhynchites amboinensis* was dropped because this mosquito species is not a blood-feeder and is known to play no role in human transmission.

Mosquito species-pathogen relationships were scored from 1 to 5 based on the strongest evidence for the relationship across available studies: (1) successful infection of females under laboratory conditions (lab infection), (2) successful dissemination of the pathogen within the vector in the laboratory (lab dissemination), (3) successful transmission from infected vector to an uninfected organism under laboratory conditions (lab transmission), with one additional point scored if the pathogen was documented to be isolated from a wild-caught adult female mosquito (wild infection). Widely agreed upon vectors of a specific pathogen in humans, as identified in more than one primary or secondary source as a known vector, were scored the highest possible strength of evidence, 5. Each mosquito–pathogen pair was assigned the maximum score across all documented evidence, such that, for example, a mosquito documented to have been lab infected in one study and lab-disseminated in another would be assigned a maximum score of 2, based on the strongest documented relationship. Note that the final assigned score for a mosquito–pathogen relationship may be obtained in more than one way. For instance, a species could be scored as a 1 either because it is documented as lab infected or wild infected; a 2 either because it is known to be infected in the wild and in the lab (1 + 1) or because it shows dissemination in the lab (2) and thus is also assumed to have been lab infected; and a 3 either because it is known to be infected in the wild and lab-disseminated (1 + 2) or because it shows transmission in the lab (3) and thus is also assumed to have been lab infected and disseminated. The two highest categories were from known vectors (5), or successful laboratory transmission and documented wild infection (3 + 1). Scores were used as importance weights for each vector–pathogen pair in subsequent network analysis.

We used networks to describe the relationships between mosquito vectors and pathogens in terms of nodes that symbolize each mosquito species or pathogen, and edges that represent evidence for a potential or known relationship between a mosquito vector and a pathogen. Network edges were weighted according to the evidence score (1–5) assigned to each vector–pathogen pair. We compared the full network of all potential vector–pathogen associations to a network that included only known vector–pathogen associations (i.e., score of 5). For each network, we used the Louvain clustering algorithm to group vectors and pathogens into “communities” by optimizing modularity, which measures the density of links within the community relative to outside the community [15]. We characterized each network structure as the degree to which clusters were connected, nested, or isolated from other nodes by calculating measures of nestedness and modularity. The nestedness metric based on overlap and decreasing fill (NODF) estimates how many nodes are connected to other nodes, with values of 0 indicating non-nestedness, 100 perfect nesting, and 50 random associations [16]. The mean standard deviation within clusters is a straightforward estimate of modularity that ranges between zero and one [17]. Modularity measures, “the tendency of a network to be compartmented into separated clusters of interacting nodes” [17]. These two values have been shown to be inversely correlated although not perfectly so [18]. Node centrality, calculated as the betweenness centrality index (BCI), is a measure of how pivotal each node is to the network, in particular as bridges between nodes or clusters, and has been proposed as a measure of generalists in pollinator networks [19]. Computations were carried out with the package “igraph” in R (www.r-project.org). Measures of network structure were calculated using the package “bipartite”.

## Results

Our analysis identified 894 mosquito–pathogen pairs from 78 disease-causing pathogens (Additional file 1: Table S1). Of the described 3586 mosquito species (mosquito-taxonomic-inventory info/valid-species-list/), 331 (9.2%) were identified as having some relationship to at least one pathogen, with 243 (6.8%) species showing wild or laboratory infection, laboratory dissemination, or transmission, and 88 (2.5%) identified as known vectors (Additional file 1: Table S1). Of the 78 pathogens, 28 have known vectors, whereas 50 did not (Additional file 1: Table S1). Of the 331 total vector species, 76% belonged to three genera (98 species of *Aedes*, 85 *Anopheles*, and 68 *Culex*), with 20 other genera adding between one and 13 species. The majority of the 78 identified pathogens were arboviruses, in the families Bunyaviridae (40), Flaviviridae (17), Togoviridae (15), Reoviridae (2), and Picornaviridae (1). Besides viruses, pathogens also included bacteria (Francisellaceae), *Plasmodium* spp. (malaria), and nematodes (Filaridae, Onchocercidae) (Additional file 1: Table S1).

**Fig. 1:**
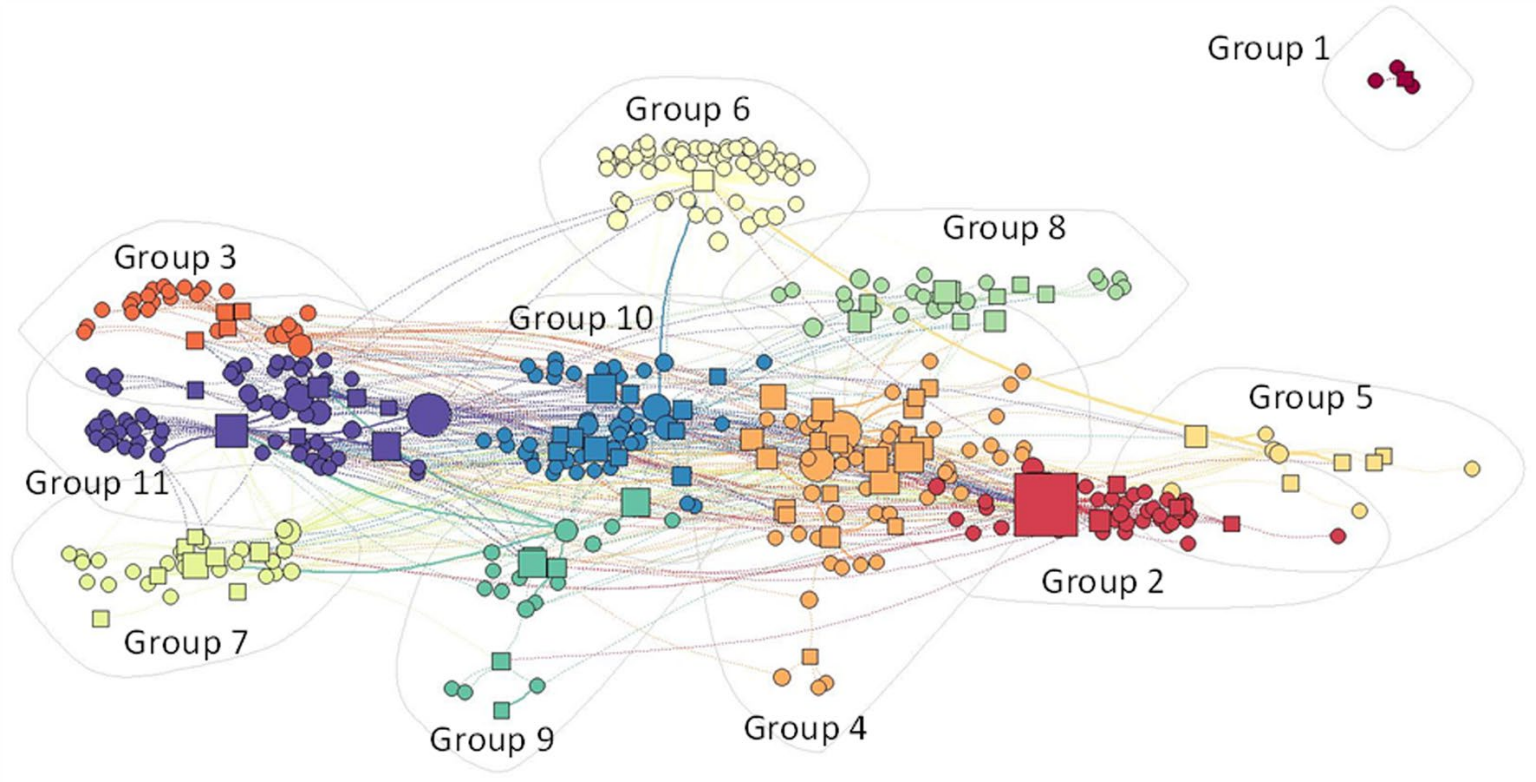
Full pathogen and vector network illustrating vectors and pathogens within each cluster (color-coded and outlined in light gray). The relative size of the shape indicates the betweenness centrality index (BCI) score in the full network, with circles indicating vectors and squares indicating pathogens. Larger symbols indicate higher BCI values. Lines connect vectors and pathogens, with known vectors/pathogen relationships connected by solid lines, and potential vector/pathogen relationships with dashed lines

The full network included 894 edges (mosquito–pathogen pairs) and 419 nodes, representing the 78 disease-causing pathogens and 331 likely or known vectors (Fig. 1). The Louvain clustering algorithm identified eleven clusters (Additional file 2: Table S2). Only one cluster (cluster 1) was not connected to any others, reflecting a potential group of specialists (*Taeniorhynchus* sp.) uniquely capable of transmitting mengovirus. All other clusters were connected by one or more generalist vectors potentially capable of transmitting many different types of pathogens. Vectors tended to cluster with pathogens of similar type, indicating that vectors with the ability to transmit one type of virus may be able to transmit other pathogens of that type. In particular, cluster 4 contained vectors of hemorrhagic arboviruses (e.g., dengue, yellow fever), whereas cluster 11 contained vectors of several encephalitis-causing viruses (e.g., West Nile, St. Louis, western equine).

For clusters and their vector–pathogen associations, the most complex clusters included cluster 4 (20 pathogens), cluster 10 (11 pathogens), cluster 8 (nine pathogens), and cluster 11 (seven pathogens). Other clusters ranged from one to six pathogens. Cluster 4 was dominated by *Aedes* (29 species) and *Haemogogus* (six species) and contained multiple arboviruses, including many considered hemorrhagic or those that cause high fever and severe joint pain (e.g., Ross River, Mayaro, yellow fever, dengue, chikungunya). This cluster also linked to two of the most important vectors, *Aedes aegypti* and *Aedes albopictus*. Cluster 10 contained 13 species of *Culex*, and 11 *Aedes,* as well as one to six species of several other genera. Of the 10 pathogens (all viruses) in this cluster, eight were bunyaviruses. Cluster 8 had a wide range of genera, with seven species of *Culex* dominating, and eight other genera of 1–3 species each. Cluster 7 included 24 mosquito species, of which most (17) were *Aedes*, including the widespread *Aedes vexans*. Of the eight pathogens identified, seven were viruses belonging to several genera, and *Francisella tularensis*, the bacteria that causes Tulmermia, one of the only bacterial pathogens known to be transmitted by mosquitoes. Cluster 6 was centered exclusively on malaria and as such contained 59 species of *Anopheles*; however, it was also connected via some species to other clusters (e.g., clusters 2, 5, 10). Cluster 11, with 58 vectors, had several pathogens that cause symptoms involving the nervous system (Japanese encephalitis, St. Louis encephalitis, West Nile virus), most of which have *Culex* as vectors. This cluster also contained the most connected mosquito in the network, *Culex quinquefasciatus*. Besides several arboviruses, cluster 5 (six mosquito species) included lymphatic filariasis, transmitted via a nematode, which is the causative agent of elephantiasis.

Network centrality is a measure of how connected each node is to other nodes in the network [20]. A mosquito node with high centrality is capable of transmitting many pathogens that may also be transmitted by many other mosquitos in the network, and thus helps maintain pathogen circulation from vector to host to other vectors within the network. The top three vectors based on the BCI were *Cx. quinquefasciatus* (BCI = 0.092, 27 pathogen associations), *Ae. aegypti* (BCI = 0.086, 38 pathogens associations), and *Ae. albopictus* (BCI = 0.065, 24 pathogen associations). Other species in the top 12 (*Culex tarsalis*, *Anopheles quadramaculatus*, *Aedes vexans*, *Culex nigripalpus*, *Culex taeniopus*, *Psorophora ferox*, *Culex pipiens*, *Anopheles coustani*, *Aedes triseriatus*) had BCI values about half that of the three top species (ranging from 0.020 to 0.043), with 190 species having BCI ~ 0.

**Fig. 2:**
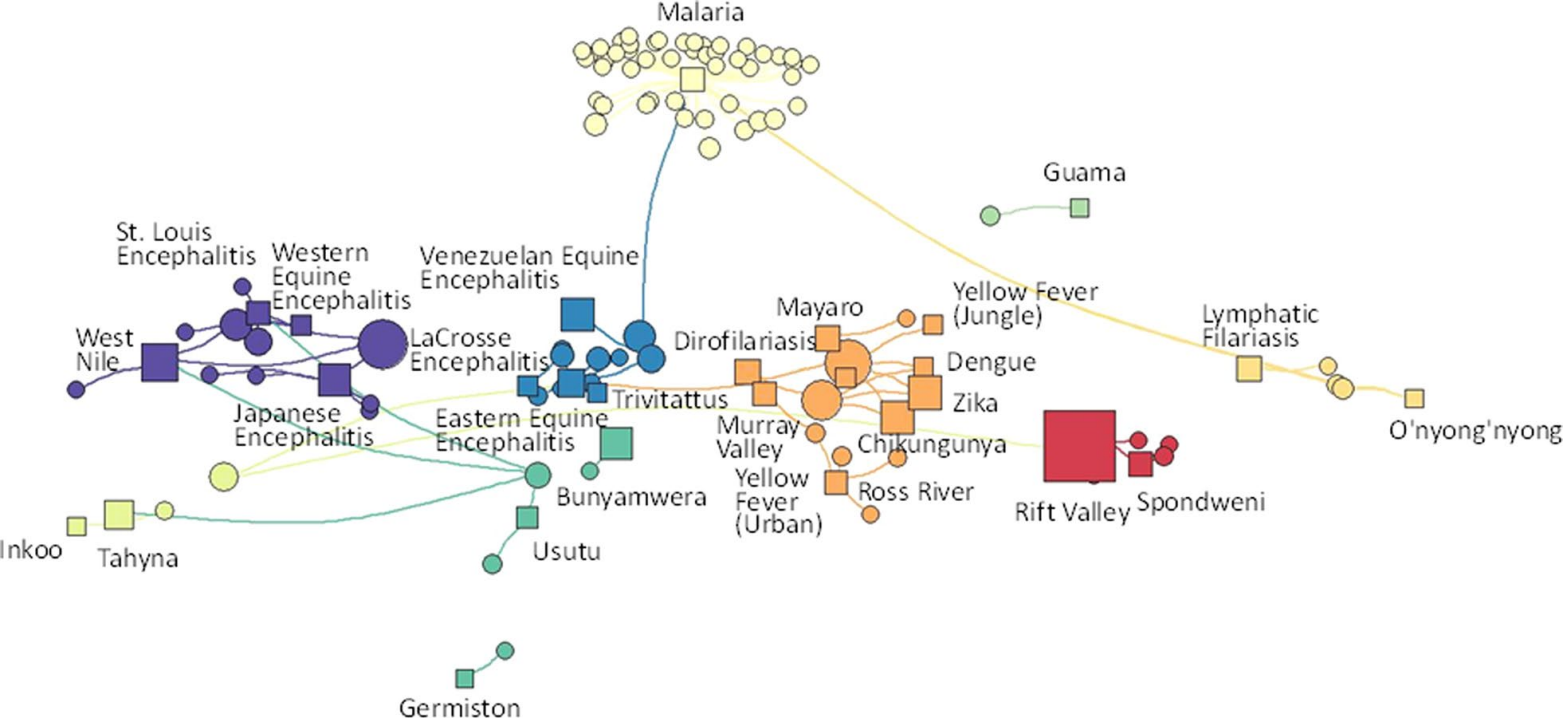
Subset of the full pathogen and vector network (as visualized in Fig. 1), showing the placement of known vectors and their pathogens. The relative size of the shape indicates the BCI score in the full network, with circles indicating vectors and squares indicating pathogens (labeled)

The network restricted to known vectors included 28 pathogens and 88 mosquito species as nodes with 108 mosquito–pathogen pairs (Fig. 2), distributed into 15 clusters by the clustering algorithm (Additional file 1: Table S1). In contrast to when all associations were included, there were few connections among clusters, with only two sets of clusters sharing vectors [clusters 7 (malaria) with 9 (Spondweni virus) and 14 (eastern equine encephalitis, Rift Valley fever, Venezuelan equine encephalitis) with 15 (chikungunya, dengue, dirofilariasis, jungle yellow fever, urban yellow fever urban, and Zika virus)].

**Fig. 3:**
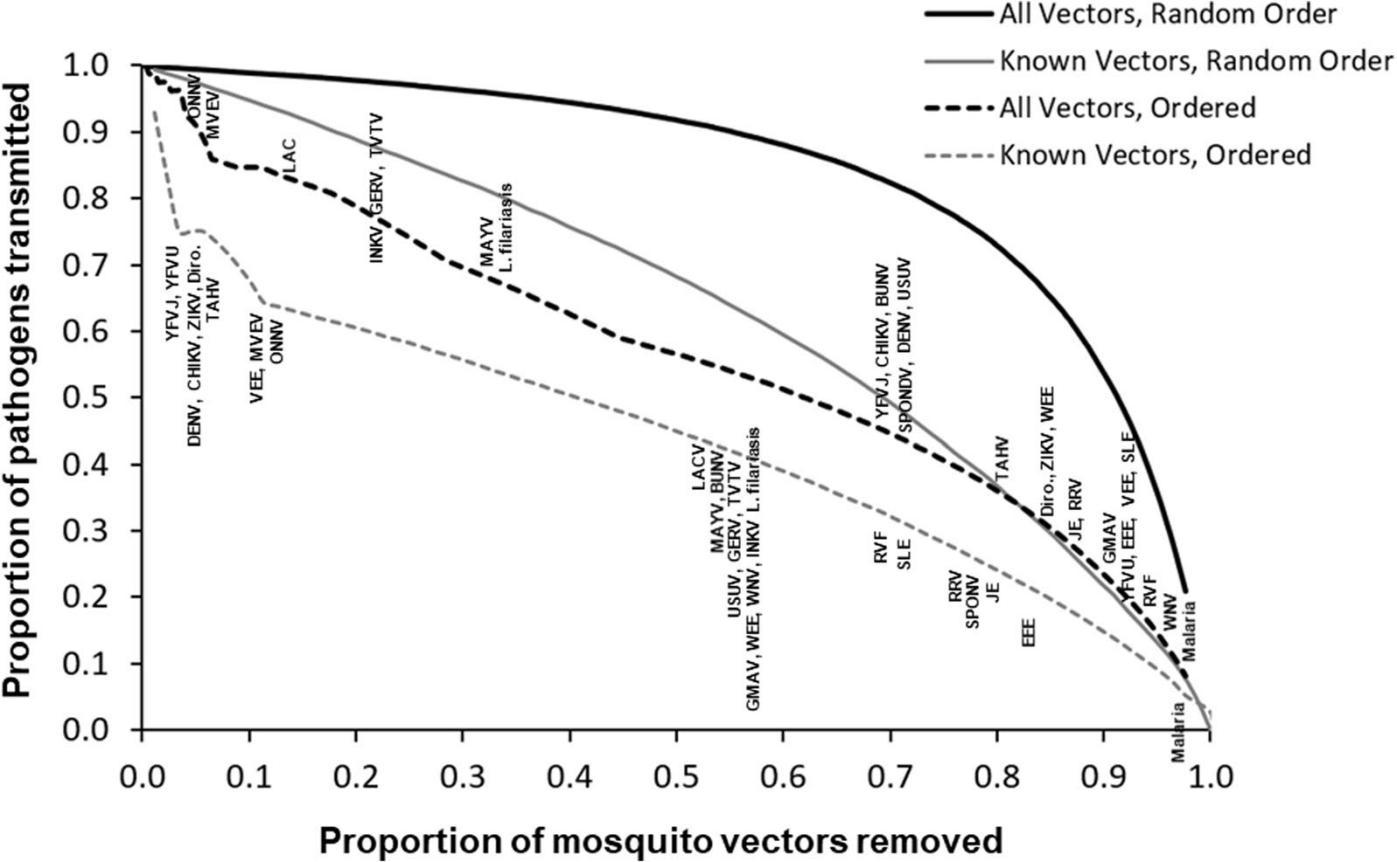
Extinction curves for the network with all vector and pathogen combinations (black lines), or only those of known vectors (gray lines) indicating the proportion of pathogens that remain transmitted as mosquito vectors are either randomly removed (solid lines) or removed in order from most to least connected (dashed lines). For the ordered removals, individual pathogens (abbreviations) are placed on the ordered lines for all vectors and known vectors where those pathogens would be removed (extinctions) from the network after removing vectors

Nestedness and modularity were used to evaluate the degree to which clusters were connected or were isolated from other nodes in the full model (Fig. 1) as compared with the model of known vectors (Fig. 2). When only known vectors were considered, the mosquito–pathogen network was highly compartmentalized (NODF = 3.71; modularity = 0.73), with clusters moderately isolated from each other and minimal connections among clusters. However, the degree of redundancy among clusters increased and the degree of compartmentalization decreased when the full network of possible vectors was considered (NODF = 8.77; modularity = 0.67), indicating that additional potential vectors were not simply added to each cluster uniformly, but instead often formed connections, or bridges, among clusters. The robustness of mosquito–pathogen associations in each network was then evaluated by estimating extinction curves as the number of pathogens that become disconnected from the network as mosquito vectors were removed, one by one, either randomly or in order of most connected to least connected using BCI values [21]. Vector extinction in the network, and secondary extinction of associated pathogens, thus essentially represents long-term suppression of the vector population to the point where the reproductive rate of each pathogen for human infections is decreasing toward zero. When mosquito species were randomly eliminated in the restricted model of known vectors, it required the elimination of on average 6.4 species to “break” the network, such that a pathogen was disconnected and left without a vector (Fig. 3, solid gray line). However, in the full network model when all possible associations were considered, it took an average of 41.1 mosquito species to be eliminated before a single pathogen was disconnected from the network (Fig. 3, solid black line). In fact, under this scenario, it would require > 90% of mosquito species to be removed to eliminate 50% of all pathogens. Robustness was calculated as the area under the extinction curve (0.0–1.0, 22), and was high for both the full network (0.84) and the known vector network (0.63) when mosquito vectors were randomly removed. For the known vector network, if the most connected mosquito vectors were preferentially targeted for elimination, the known vector network showed some instability with a robustness value < 0.5 (0.38), such that removal of mosquito vectors led to a greater than one-to-one removal of pathogens (Fig. 3, dashed gray line). But when possible and likely vectors were also included in the network, robustness increased (0.56, Fig. 3 dashed black line).

**Fig. 4:**
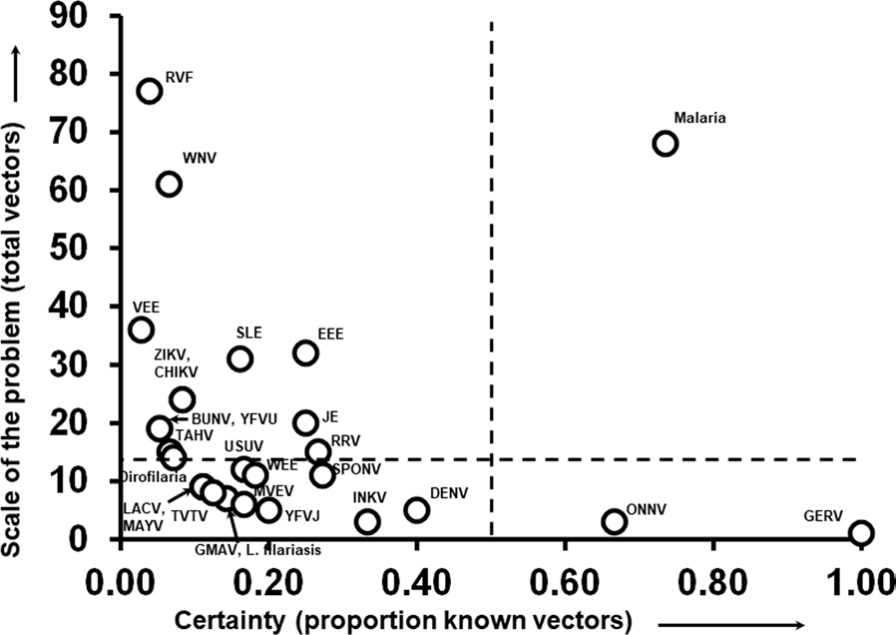
Relationship for individual pathogens of human medical importance between the total number of vectors with any association (scale of the problem) and the proportion of those that are known vectors (certainty). The dashed line for the *y*-axis was based on the median number of total vectors per pathogen (13), whereas the dashed line in the *x*-axis was placed at 0.5 to demarcate pathogens with either less than half or more than half of the vectors considered as known vectors

Finally, for a given pathogen, the difference between our current certainty about known vectors (i.e., the proportion of potential identified vectors that are known to be vectors) and the scale of the problem (i.e., the total number of potential vectors) is indicative of the level of challenge being faced to eradicate these pathogens through mosquito control (Fig. 4). Pathogens with few total vectors and a high proportion of known vectors (low scale of problem, high certainty, lower right quadrant of Fig. 4) may be relatively easy to eliminate with targeted eradication efforts. However, there were only two pathogens in this category (Germiston, O'nyong'nyong), both of which cause relatively few annual human cases. Pathogens with few overall vectors but with more uncertainty (lower left quadrant, Fig. 4) may be manageable with additional research to improve our understanding of known vectors. This category includes many important pathogens, including dirofilariasis, lymphatic filariasis, La Crosse encephalitis, Mayaro, and dengue. Finally, the upper two quadrants highlight pathogens where the scale of the problem is relatively large, and where control efforts may be more daunting given the number of potential species involved. Pathogens with a large scale of problem and low certainty (upper left quadrant, Fig. 4), represent the greatest challenges, as these have a higher redundancy of species and few species that are obvious targets of suppression. This group contains problematic and widely dispersed pathogens like West Nile virus, chikungunya, Zika, and urban yellow fever (Fig. 4). The only pathogen we identified as high certainty and a large-scale problem was malaria, which contains many known vectors (and pathogen species) and many others likely implicated (all *Anopheles*).

## Discussion

Our work points out that mosquitoes of human medical importance are rare among the Culicidae, with less than 10% having any known or potential role in disease transmission. However, this is probably an underestimate, for a number of reasons. First, other unidentified species may have the capacity to be important for human disease transmission but are understudied. For example, Evans et al. [5] predicted that > 25 species of mosquitoes could be important for the transmission of Zika worldwide, but noted that most control efforts for managing outbreaks were focused on only two (*Ae. aegypti* and *Ae. albopictus*); our analysis identified 25 species in total. For neglected and obscure pathogens, an underestimate is almost certainly true, especially in areas of the world where vector surveillance is underfunded or non-existent. Second, other mosquito species likely exist with respect to importance in disease cycles. Specifically, our analyses do not consider mosquitoes involved in zoonotic cycles (e.g., West Nile virus, eastern equine encephalitis) that do not bite humans. This could make the issue of eradication even greater if one considers that these other species act as reservoir vectors to maintain those pathogens outside of humans. Thus, even with targeted suppression of the known human vectors, the pathogen may still remain in the environment for introduced species of those experiencing range expansion to transmit.

By design, our analysis did not consider the geographical range of vectors or pathogens as a factor in the network, as we were focused on understanding global patterns of vector–pathogen associations and global suppression of pathogens. However, several prominent vectors have worldwide or nearly worldwide distributions, including the three species with the highest BCI (*Cx. quinquefasciatus*, *Ae. aegypti*, *Ae. albopictus*). Ultimately, the robustness of individual mosquito–pathogen associations is likely dependent on vector ecology and behavior, in particular local factors, such as the overlapping presence of resident mosquito species and the pathogen(s), land-use and environmental variation, as well as the population density and composition of human hosts and host preference [22]. Thus, we might expect that local eradication of vectors (and thus pathogens) may be a more manageable goal than suppression of the several common worldwide invasive species. However, this could be complicated by several factors, including government interest in and funding of eradication and the logistics of mosquito suppression. As much as focusing on vector–pathogen networks at a narrower geographical scale could make these findings more meaningful, this approach would also belie the fact that pathogens can jump into new hosts or expand their range due to a number of circumstances. For instance, Zika saw a rapid expansion out of Africa in the last seven years into several new continents, with devastating effects on human hosts [4]. Such expansion is unpredictable, but should novel pathogens expand into areas where existing vectors reside, it can cause significant outbreaks among naive human hosts. Thus, having a more inclusive global perspective, like the one we use here, is potentially more useful in these cases.

Another take-away from our analysis is that focusing on a single mosquito would not greatly affect network stability, given the high degree of redundancy in vectors for each pathogen. Removing the most connected vector, *Cx. quinquefasciatus*, would leave > 90% of the network intact. Notably, our approach considered two extreme possibilities of vector–pathogen relationships: known vectors only versus all potential vectors at a global scale. Reality, however, likely lies somewhere in-between these two possibilities and considering a general lack of knowledge about the specific role of many of these vectors in humans, including overlaps in geographical distribution, invasion potential, effective population size, or biting rates toward human hosts, we suggest it lies closer to results including all potential vectors. Current strategies to target specific known mosquito vectors (e.g., *Aedes aegypti*, *Anopheles gambiae*) for elimination to reduce pathogen transmission may be inadequate, especially with the presence of potential or likely vectors that can create redundancies in the global mosquito–vector network. Potential vectors, particularly those with high connectedness in mosquito–pathogen networks, warrant further investigation to better understand their roles in human disease transmission, their potential for introducing pathogens to novel geographical areas, and their need to be integrated into pest management strategies. Although mosquitoes of medical importance are rare among Culicidae, they remain the greatest global threat to human health.

## Conclusion

Mosquitoes that transmit pathogens to humans are rare among the Culicidae, accounting for between 2.5 and 9.3% of all species, with most concentrated within three genera (*Aedes*, *Anopheles*, *Culex*). Although rare, mosquitoes of human medical importance, along with 78 disease-causing pathogens, support a robust network that appears to be resilient to elimination of both specific and random mosquito species. This inherent robustness is likely a main reason why it remains difficult to eliminate specific pathogens, like dengue, yellow fever, and malaria, across the world. Future work may examine smaller geographically restricted networks, as it is at these smaller scales that elimination of pathogens is likely, and where targeted mosquito control efforts will have higher success. Our findings however also point to deficiencies in our understanding of the specific role of all mosquito species in transmitting pathogens to humans and add to the urgency of our attempts to understand both the past, present, and future role of mosquitoes in vector-borne disease outbreaks [2].

## Supplementary Information

**Additional file 1: Table S1.** Mosquito vectors associated with pathogens of human disease relevance. For each pathogen/disease (with abbreviation), species of mosquito that fall into five categories are listed. Wild infection are those that have been found to carry the virus during sampling of mosquitoes collected in nature, Lab infection are those who were positive for a virus after being offered an infectious blood meal, Lab Dissemination are those that showed replication of the virus in tissue (e.g., legs), Lab Transmit were those that could pass the pathogen on to a host under laboratory conditions (often to a non-human mammal), and Known Vectors were those that were considered to be a central species in maintaining the pathogen in nature and directly infecting humans. In all cases, we assumed species names were used as sensu stricto (e.g., *Anopheles gambiae*) based on the publications that listed them. We cannot know for sure in all cases as many publications did not list s.s. or s.l., but given the nature of those publications, we assumed they were s.s.

**Additional file 2: Table S2.** Associations of vectors and pathogens from cluster analysis (Figs. 1, 2). Groups identified from analysis of known vectors only are identified in bold, with superscripts corresponding to cluster number.

## Data Availability

All data are available in the main text or the Additional files.

## References

[1] Carter R, Mendis KN (2002). Evolutionary and historical aspects of the burden of malaria. Clin Microbiol Rev.

[2] Althi TS, Shocket MS, Couper LI, Nova N, Caldwell IR, Caldwell JM (2021). The influence of vector-borne disease on human history: socio-ecological mechanisms. Ecol Let.

[3] Löwy I (2017). Leaking containers: success and failure in controlling the mosquito *Aedes aegypti* in Brazil. Am J Public Health.

[4] Braak L, de Gouveia Almeida AP, Cornel AJ, Swanepoel R, de Jager C (2018). Mosquito-borne arbovirus of African origin: review of key viruses and vectors. Parasit Vectors..

[5] Evans MV, Dallas TA, Han BA, Murdock CC, Drake JM (2017). Data-driven identification of potential Zika virus vectors. Elife.

[6] Bellekom B, Hackett TD, Lewis OT (2021). A network perspective on the vectoring of human disease. Trends Parasit..

[7] Estrada-Peña A, de la Fuente K, Ostfeld RS, Cabezas-Crus A (2015). Interactions between tick and transmitted pathogens evolved to minimise competition through nested and coherent networks. Nat Sci Rep.

[8] Gómez JM, Nunn CL, Verdú M (2013). Centrality in primate-parasite networks reveals the potential for the transmission of emerging infectious diseases to humans. Proc Nat Acad Sci.

[9] Stephens CR, Heau JG, González C, Ibarra-Cerdeña CN, Sánchez-Cordero V (2009). Using biotic interaction networks for prediction in biodiversity and emerging diseases. PLoS ONE.

[10] Librán-Embid F, Grass I, Emer C, Ganuza C, Tscharntke T (2021). A plant–pollinator metanetwork along a habitat fragmentation gradient. Ecol Let.

[11] Beran GW (2017). Handbook of Zoonoses, Section B: Viral Zoonoses.

[12] Beaty BJ, Marquardt WC (1996). The biology of disease vectors.

[13] Theiler M, Downs WG (1973). The arthropod-borne viruses of vertebrates: an account of the Rockefeller Foundation Virus Program, 1951–1970.

[14] de Oliveira Filho EF, Carneiro IO, Ribas JRL, Fisher C, Marklewitz M, Junglen S (2020). Identification of animal hosts of Fort Sherman virus, a New World zoonotic orthobunyavirus. Trans Emer Dis.

[15] Blondel VD, Guillaume J-L, Lambiotte R, Lefebvre E (2008). Fast unfolding of communities in large networks. J Stat Mech.

[16] Almeida-Neto M, Guimaraes P, Guimaraes PR, Loyola RD, Ulrich W (2008). A consistent metric for nestedness analysis in ecological systems: reconciling concept and measurement. Oikos.

[17] Strona G, Veech JA (2015). A new measure of ecological network structure based on node overlap and segregation. Method Ecol Evol.

[18] Fortuna MA, Stouffer DB, Olesen JM, Jordano P, Mouillot D, Krasnov BR (2010). Nestedness versus modularity in ecological networks: two sides of the same coin?. J Anim Ecol.

[19] Martín Gonzáles AM, Dalsgaard B, Olesen JM (2010). Centrality measures and the importance of generalist species in pollination networks. Ecol Complex.

[20] Hurst CJ, Hurst CJ (2018). The connections between ecology and infectious disease. Advances in environmental microbiology.

[21] Memmott J, Waser NM, Price MV (2004). Tolerance of pollination networks to species extinctions. Proc Royal Soc B.

[22] Takken W, Verhulst NO (2013). Host preference of blood-feeding mosquitoes. Ann Rev Entomol.

