# Supplementary material for: Robust network stability of mosquitoes and human pathogens of medical importance
Source: Parasit Vectors. 2022 Jun 20;15:216. doi: 10.1186/s13071-022-05333-4 (PMC9208160; doi:10.1186/s13071-022-05333-4)
Supplement: Supplementary file 1 — Additional file 1: Table S1. Mosquito vectors associated with pathogens of human disease relevance. For each pathogen/disease (with abbreviation), species of mosquito that fall into five categories are listed. Wild infection are those that have been found to carry the virus during sampling of mosquitoes collected in nature, Lab infection are those who were positive for a virus after being offered an infectious blood meal, Lab Dissemination are those that showed replication of the virus in tissue (e.g., legs), Lab Transmit were those that could pass the pathogen on to a host under laboratory conditions (often to a non-human mammal), and Known Vectors were those that were considered to be a central species in maintaining the pathogen in nature and directly infecting humans. In all cases, we assumed species names were used as sensu stricto (e.g., Anopheles gambiae) based on the publications that listed them. We cannot know for sure in all cases as many publications did not list s.s. or s.l., but given the nature of those publications, we assumed they were s.s. [file 13071_2022_5333_MOESM1_ESM.docx]

| Pathogen/ disease (abbreviation) | Taxonomy | Known vector | Lab transmission | Lab dissemination | Lab infection | Wild infection |
| --- | --- | --- | --- | --- | --- | --- |
| Apeu (APEUV) | Family Bunyaviridae  Genus Buynyavirus |  |  | *Aedes aegypti* [1]  *Culex quinquefasciatus* [1] |  | *Aedes arborealis* [1]  *Aedes septemstriatus* [1]  *Culex aikenii* [1] |
| Banzi (BANV) | Family Flaviviridae  Genus Flavivirus |  | *Culex neavei* [2] |  |  | *Culex rubinotus* [1,3]  *Mansonia africana* [3]  *Aedes caspius* [4]  *Culex hortensis* [4] |
| Banna (BAV) | Family  Reoviridae  Genus  Seadornavirus |  |  |  |  | *Culex annulus* [5]  *Culex tritaeniorhynchus* [5] |
| Barmah Forest (BFV) | Family  Togaviridae  Genus  Alphavirus |  | *Aedes notoscriptus* [6]  *Aedes procax* [7]  *Aedes vigilax* [7, 8] | *Aedes vigilax,* [8] |  | *Coquillettidia linealis* [1] |
| Bunyamwera virus (BUNV) | Family  Bunyaviridae  Genus  Bunyavirus | *Aedes quasiunivittatus* [9] | *Aedes ochraceus* [10]  *Aedes luteocephalus* [10,11]  *Aedes quasiunivittatus* [9]  *Anopheles quadrimaculatus* [10]  *Culex pipiens* [11]  *Culex quinquefasciatus* [12]  *Culex univittatus* [12] | *Aedes luteocephalus* [11]  *Culex pipiens* [11] | *Aedes luteocephalus* [11]  *Culex pipiens* [11]  *Culex univittatus* [11] | *Aedes aegypti* [13]  *Aedes albopictus* [13]  *Aedes canadensis* [13]  *Aedes circumleteolus* [13]  *Aedes luteocephalus* [14]  *Aedes mcintoshi* [11]  *Aedes quasiunivittatus* [15]  *Aedes* sp. [16]  *Aedes triseriatus* [17]  *Anopheles funestes* [18]  *Anopheles gambiae* [9, 19*,*20]  *Culex pipiens* [14]  *Culex univittatus* [14]  *Limatus asulleptus* [21]  *Mansonia titillans* [22]  *Psorophora albigenu* [22]  *Psorophora ferox* [22] |
| Bussuquara (BSQV) | Family  Flaviviridae  Genus  Flavivirus |  |  | *Aedes aegypti* [1]  *Culex quinquefasciatus* [1] |  | *Coquillettidia venezuelensis* [1]  *Culex crybda* [23]  *Culex taeniopus* [1]  *Culex vomerifer* [1]  *Mansonia titillans* [1]  *Trichoprosopon* [1]  *Culex* sp*.* [1, 24] |
| Bwamba (BWAV) | Family  Bunyaviridae  Genus  Bunyavirus |  |  | *Aedes aegypti* [1]  *Anopheles quadrimaculatus* [1]  *Culex quinquefasciatus* [1] | *Aedes aegypti* [25]  *Anopheles quadrimaculatus* [25]  *Culex pipiens* [25] | *Anopheles funestus* [1, 25, 26]  *Anopheles gambiae* [1]  *Aedes circumluteolus* [25]  *Anopheles coustani* [25]  *Anopheles furcifer* [25]  *Anopheles gambiae* [25]  *Mansonia uniformis* [25] |
| Cache Valley virus (CVV) | Family  Bunyaviridae  Genus  Bunyavirus |  | *Aedes sollicitans* [27]  *Aedes taeniorhynchus* [27]  *Anopheles quadrimaculatus* [28]  *Coquillettidia perturbans* [28] | *Culiseta inornata* [29] | *Aedes dorsalis* [29]  *Aedes*  *melanimon* [29]  *Aedes nigromaculis* [29]  *Culex tarsalis* [29] | *Aedes albopictus* [29]  *Aedes canadensis* [31]  *Aedes cantator* [31]  *Aedes cinereus* [31]  *Aedes japonicus* [31]  *Aedes sollicitans* [31, 32]  *Aedes taeniorhynchus* [29*,*31*,*33]  *Aedes triseriatus* [31]  *Aedes trivittatus* [31, 34]  *Aedes vexans* [31, 34]  *Anopheles crucians* [32]  *Anopheles grabhamii* [29]  *Anopheles punctipennis* [31, 34]  *Anopheles quadrimaculatus* [31, 34, 35]  *Anopheles walkeri* [31]  *Coquillettidia perturbans* [31, 34]  *Culex salinarius* [31]  *Culiseta inornata* [34, 36*,* 37]  *Culiseta melanura* [31]  *Psorophora columbiae* [34]  *Psorophora confinnis* [38]  *Psorophora ferox* [31] |
| California Encephalitis (CE) | Family Bunyaviridae  Genus  Bunyavirus |  | *Aedes aegypti* [39, 40]  *Aedes dorsalis* [41]  *Aedes melanimon* [42] |  | *Aedes aegypti* [39]  *Aedes dorsalis* [41]  *Aedes varipalpus* [41]  *Culex tarsalis* [39, 41]  *Culiseta inornata* [41] | *Aedes melamion* [43]  *Aedes nigromaculis* [44]  *Culex dorsalis* [42]  *Culex tarsalis* [42]  *Psorophora signipennis* [44] |
| Caraparu Virus (CARV) | Family Bunyaviridae  Genus  Bunyavirus |  | *Aedes aegypti* [45]  *Culex quinquefasciatus* [1] |  |  | *Aedes scapularis* [24]  *Aedes serratus* [24]  *Culex portesi* [46]  *Culex vomerifer* [46] |
| Catu virus (CATUV) | Family  Bunyaviridae  Genus  Bunyavirus |  | *Culex portesi* [1] |  |  |  |
| Chikungunya (CHIKV) | Family  Togaviridae  Genus  Alphavirus | *Aedes aegypti* [47]  *Aedes albopictus* [48] | *Aedes aegypti* [47]  *Aedes albopictus* [47]  *Aedes calceatus* [49]  *Aedes notoscriptus* [50]  *Aedes polynesiensi* [51]  *Aedes procax* [50]  *Aedes togoi* [49]  *Aedes triseriatus* [49]  *Aedes vigilax* [50]  *Anopheles albimanus* [49]  *Coquillettidia linealis* [50]  *Eretmapodites chrysogaster* [49] | *Aedes aegypti* [47]  *Aedes albopictus* [47]  *Aedes hensilli* [52]  *Aedes notoscriptus* [50]  *Aedes procax* [50]  *Aedes vittatus* [53, 54]  *Aedes vigilax* [50]  *Coquillettidia linealis* [50] | *Aedes fulgens* [52, 55]  *Aedes vittatus* [53]  *Coquillettidia linealis* [50] | *Aedes africanus* [1, 56]  *Aedes dalzieli* [57]  *Aedes furcifer* [57]  *Aedes luteocephalus* [57]  *Culex gelidus* [58]  *Culex quinquefasciatus* [58]  *Culex tritaeniorhynchus* [58]  *Mansonia uniformis* [56]  *Mansonia fuscopennata* [56] |
| Dengue (DENV) | Family  Flaviviridae  Genus Flavivirus | *Aedes aegypti* [48]  *Aedes albopictus* [48] | *Aedes scutellaris* [49] | *Aedes aegypti* [59, 60]  *Aedes albopictus* [60]  *Aedes vittatus* [61] | *Aedes aegypti* [62]  *Aedes mediovittatus* [62] | *Aedes aegypti* [63*,* 64]  *Aedes albopictus* [64] |
| Dirofilariasis | Family Onchocercidae  Genus Dirofilaria | *Aedes albopictus* [65] |  |  | *Aedes aegypti* [66]  *Aedes albifasciatus* [66]  *Mansonia titillans* [66]  *Psorophora cyanescens* [66] | *Aedes albifasciatus* [66]  *Aedes albopictus* [67*,* 68]  *Aedes caspius* [68]  *Aedes vexans* [68]  *Anopheles maculipennis* [68, 69, 70]  *Coquillettidia richiardii* [68, 70]  *Culex dolosus* [66]  *Culex pipiens* [66, 67, 68, 70]  *Culex theileri* [70, 71]  *Ochlerotatus crinifer* [67]  *Psorophora ferox* [67] |
| Eastern Equine Encephalitis (EEE) | Family Togaviridae  Genus Alphavirus | *Aedes aegypti* [71]  *Aedes canadensis* [72]  *Aedes sollicitans* [72]  *Aedes taenorhynchus* [73]  *Aedes vexans* [71, 72, 74]  *Coquillettidia perturbans* [72]  *Culex pedroi* [73]  *Culex taeniopus* [75]  *Coquillettidia perturbans* [31] | *Aedes aegypti* [71]  *Aedes albopictus* [76]  *Aedes atropalpus* [71]  *Aedes canadensis* [77]  *Aedes vexans* [77]  *Anopheles punctipennis* [77]  *Anopheles quadrimaculatus* [77]  *Coquillettidia perturbans* [77]  *Culex salinarius* [77]  *Aedes cantator* [71]  *Aedes sollicitans* [71]  *Aedes taeniorhynchus* [71]  *Aedes triseriatus* [71]  *Aedes vexans* [71] |  | *Aedes aegypti* [71]  *Aedes albopictus* [76]  *Aedes atropalpus* [71]  *Aedes cantator* [71]  *Aedes sollicitans* [71]  *Aedes triseriatus* [71]  *Aedes vexans* [71]  *Anopheles punctipennis* [71]  *Culex pipiens* [1, 71]  *Culex salinarius* [1, 71]  *Mansonia perturbans* [1, 71] | *Aedes albopictus* [1]  *Aedes canadensis* [72]  *Aedes cantator* [72]  *Aedes cinereus* [72]  *Aedes sollicitans* [31]  *Aedes trivattatus* [72]  *Aedes triseriatus* [72]  *Aedes vexans* [72, 74, 78]  *Anopheles punctipennis* [72]  *Anopheles quadrimaculatus* [72]  *Anopheles walkeri* [72]  *Culex dunni* [75]  *Culex panocossa* [75]  *Culex sacchettae* [75]  *Culex restuans* [72]  *Culex salinarius* [72]  *Culex taeniopus* [79]  *Culex melanura* [72]  *Culiseta morsitans* [80]  *Psorophora ferox* [72]  *Uranotaenia sapphirina* [72]  *Coquillettidia perturbans* [74, 78]  *Culex dunni* [75]  *Culex erraticus* [74, 78]  *Culex gnomatos* [81]  *Culex panocossa* [75]  *Culex peccator* [82]  *Culex pedroi* [81]  *Culiseta melanura* [74, 78]  *Culiseta morsitans* [83]  *Psorophora albigenu* [81]  *Uranotaenia sapphirina* [74, 78] |
| Everglades (EVEV) | Family Togaviridae  Genus Alphavirus |  | *Culex cedecei* [84] |  | *Aedes triseriatus* [1]  *Culex nigripalpus* [1] | *Aedes atlanticus* [1]  *Aedes taeniorhynchus* [1]  *Aedes triseriatus* [1]  *Anopheles crucians* [1]  *Culex cedecei* [1]  *Culex nigripalpus* [1] |
| Fort Sherman (FSV) | Family Bunyaviridae  Genus Bunyavirus |  |  |  |  | No identified vectors |
| Ganjam (GANV) | Family Bunyaviridae  Genus Nairovirus |  |  |  | *Aedes aegypti* [1]  *Aedes albopictus* [1] | *Culex vishnui* [85] |
| Germiston (GERV) | Family Bunyaviridae  Genus Bunyavirus | *Culex rubinotus* [86, 87] | *Culex rubinotus* [86, 87] |  |  | *Culex rubinotus* [1] |
| Getah (GETV) | Family Togaviridae  Genus Alphavirus |  | *Aedes albopictus* [88] |  | *Aedes albopictus* [88]  *Aedes funereus* [89]  *Aedes vigilax* [90]  *Culex annulirostris* [90] | *Armigeres obturbans* [91]  *Armigeres subalbatus* [91]  *Aedes vexans* [92]  *Anopheles amictus* [93]  *Anopheles hrycanus* [1]  *Culex gelidus* [1]  *Culex tritaeniorhynchus* [93]  *Culex* sp*.* [88]  *Culex vishnui* [94] |
| Guama Virus (GMAV) | Family Bunyaviridae Genus Bunyavirus | *Culex* sp [1, 95, 96]  *Culex portesi* [73] | *Culex taeniopus* [96  *Culex vomerifer* [97] |  |  | *Aedes sp.* [98]  *Culex portesi* [98*,* 99]  *Culex vomerifer* [98]  *Limatus* sp*.* [98]  *Mansonia* sp*.* [98]  *Psorophora* sp*.* [98]  *Trichoprosopon* sp. [98] |
| Guaroa Virus (GROV) | Family Bunyaviridae Genus Bunyavirus |  |  |  | *Aedes aegypti* [1, 100*,* 101]  *Aedes triseriatus* [101]  *Anopheles quadrimaculatus* [1, 100]  *Anopheles neivai* [102]  *Culex pipiens* [100]  *Culex quinquefasciatus* [1]  *Psorophora ferox* [101] | *Anopheles neivai* [103] |
| Ilesha (ILEV) | Family Bunyaviridae Genus Bunyavirus |  |  |  |  | *Anopheles gambiae* [18] |
| Ilheus (ILHV) | Family Flaviviridae  Genus Flavivirus |  |  |  | *Aedes aegypti* [104]  *Aedes scapularis* [105]  *Aedes serratus* [104]  *Culex quinquefasciatus* [105]  *Psorophora albipes* [105]  *Psorophora ferox* [105] | *Aedes serratus* [104]  *Psorophora ferox* [105] |
| Inkoo (INKV) | Family Bunyaviridae Genus Bunyavirus | *Aedes communis* [106] |  |  |  | *Aedes communis* [106]  *Aedes hexodontus* [106]  *Aedes punctor* [106, 107]  *Ochlerotatus communis* [107] |
| Issyk-Kul (ISKV) | Family Bunyaviridae Genus  Nairovirus |  | *Aedes caspius* [108] |  |  | *Anopheles hyrcanus* [1] |
| Itaqui Virus (ITQV) | Family Bunyaviridae Genus Bunyavirus |  |  |  |  | *Aedes taeniorhynchus* [75]  *Culex pedroi* [97]  *Culex portesi* [99]  *Culex vomerifer* [75, 81, 109] |
| Jamestown Canyon (JCV) | Family Bunyaviridae Genus Bunyavirus |  |  | *Aedes dorsalis* [110]  *Aedes squamiger* [110]  *Aedes stimulans* [111] |  | *Aedes abserratus* [112]  *Aedes cantator* [112]  *Aedes cataphylla* [113]  *Aedes communis* [114]  *Aedes hexodontus* [113]  *Aedes intrudens* [114]  *Aedes provocans* [114]  *Aedes pucntor* [114]  *Aedes stimulans* [111]  *Aedes vexans* [112]  *Culiseta inornate* [115, 116] |
| Japanese Encephalitis (JE) | Family Flaviviridae  Genus Flavivirus | *Culex fuscocephala* [117]  *Culex gelidus* [117]  *Culex quinquefasciatus* [118]  *Culex vishnui* [117]  *Culex bitaeniorhynchus* [117] | *Aedes albopictus* [1] |  | *Aedes albopictus* [1]  *Anopheles tesellatus* [119]  *Culex bitaeniorhynchus* [117, 119]  *Culex fatigans* [119]  *Culex tritaeniorhynchus* [119] | *Aedes vigilax* [120]  *Aedes albopictus* [1]  *Aedes butleri* [121]  *Aedes lineatopennis* [121]  *Anopheles barbirostris* [119]  *Anopheles hyrcanus* [119]  *Anopheles subpictus* [119]  *Culex annulirostris* [118]  *Culex annulus* [118]  *Culex bitaeniorhynchus* [117, 118, 119]  *Culex fuscocephala* [121]  *Culex gelidus* [121]  *Culex pipiens* [122]  *Culex quinquefasciatus* [121]  *Culex sitiens* [121]  *Culex tritaeniorhynchus* [119, 123*,* 124, 125]  *Culex vishnui* [118, 119]  *Culex whitmorei* [119]  *Culex bitaeniorhynchus* [117] |
| Kokobera (KOKV) | Family Flaviviridae  Genus Flavivirus |  |  |  | *Culex quinquefasciatus* [1] | *Aedes vigilax* [126]  *Culex annulirostris* [1] |
| LaCrosse Encephalitis (LAC) | Family Bunyaviridae  Genus Bunyavirus | *Aedes triseriatus* [127] | *Aedes aegypti* [128]  *Aedes canadensis* [128]  *Aedes japonicus* [129]  *Aedes triseriatus* [129]  *Aedes trivittatus* [128]  *Aedes vexans* [128]  *Culiseta inornata* [128] | *Aedes albopictus* [129] |  | *Aedes albopictus* [129]  *Aedes japonicus* [129]  *Aedes triseriatus* [129]  *Aedes trivittatus* [130]  *Culex pipiens* [130] |
| Lumbo Virus (LUMV) | Family Bunyaviridae  Genus Bunyavirus |  |  |  |  | *Aedes pembaensi* [131] |
| Lymphatic Filariasis  (elephantitis) | Family Filaridae  Wuchereria bancrofti, Brugia malayi, *B. timori* | *Aedes polynesiensis* [132] |  | *Aedes polynesiensis* [132]  *Anopheles funestus* [133]  *Anopheles gambiae* [133]  *Anopheles punctulatus* [134]  *Culex pipiens* [134]  *Culex quinquefasciatus* [132] | *Aedes aegypti* [132] | *Aedes polynesiensis* [132]  *Anopheles funestus* [133]  *Anopheles gambiae* [133]  *Culex quinquefasciatus* [132] |
| Madrid Virus (MADV) | Family Bunyaviridae  Genus Bunyavirus |  | *Culex vomerifer* [97] |  |  | *Culex vomerifer* [97, 101, 109]  *Culex* sp*.* [1] |
| Malaria | Family Plasmodiidae  Genus Plasmodium | *Anopheles aconitus* [135]  *Anopheles albimanus* [135]  *Anopheles albitarsis* [135]  *Anopheles annularis* [135]  *Anopheles aquasalis* [135]  *Anopheles arabiensis* [135]  *Anopheles argyritarsis* [135]  *Anopheles atroparvus* [135]  *Anopheles balabacensis* [135]  *Anopheles barbirostris* [135]  *Anopheles bellator* [135]  *Anopheles campestris* [135]  *Anopheles cruzi* [135]  *Anopheles culicifacies* [135]  *Anopheles darlingi* [135]  *Anopheles dirus* [135]  *Anopheles farauti* [135]  *Anopheles freeborni* [135]  *Anopheles flavirostris* [135]  *Anopheles fluviatilis* [135]  *Anopheles funestus* [135]  *Anopheles gambiae* [135]  *Anopheles koliensis* [135]  *Anopheles labranchiae* [135]  *Anopheles lesteri* [135]  *Anopheles letifer* [135]  *Anopheles leucosphyrus* [135]  *Anopheles maculatus* [135]  *Anopheles marajoara* [135]  *Anopheles melas* [135]  *Anopheles messeae* [135]  *Anopheles minimus* [135]  *Anopheles moucheti* [135]  *Anopheles multicolor* [135]  *Anopheles nigerrimus* [135]  *Anopheles nili* [135]  *Anopheles nuneztovari* [135]  *Anopheles pharoensis* [135]  *Anopheles pseudopunctipennis* [135]  *Anopheles pulcherrimus* [135]  *Anopheles punctimacula* [135]  *Anopheles punctulatus* [135]  *Anopheles quadrimaculatus* [135]  *Anopheles sacharovi* [135]  *Anopheles sergentii* [135]  *Anopheles sinensis* [135]  *Anopheles stephensi* [135]  *Anopheles subpictus* [135]  *Anopheles sundaicus* [135]  *Anopheles superpictus* [135] |  | *Anopheles dthali* [136]  *Culex quinquefasciatus* [137]  *Culex stigmatosoma* [137]  *Culex tarsalis* [137] |  | *Anopheles melas* [138]  *Anopheles merus* [138]  *Anopheles albimanus* [122]  *Anopheles albitarsis* [139]  *Anopheles aquasalis* [122, 139]  *Anopheles arabiensis* [138, 140]  *Anopheles atroparvus* [138, 140]  *Anopheles bellator* [122, 139]  *Anopheles brasiliensis* [139]  *Anopheles carnevalei* [141]  *Anopheles coustani* [141]  *Anopheles cruzii* [139]  *Anopheles culicifacies* [142]  *Anopheles darlingi* [139, 143]  *Anopheles dthali* [136]  *Anopheles fluviatilis* [136]  *Anopheles franciscanus* [144]  *Anopheles funestus* [138, 140]  *Anopheles gambiae* [138, 139]  *Anopheles hancocki* [138]  *Anopheles labranchiae* [138, 140]  *Anopheles marshalii* [141]  *Anopheles messeae* [135]  *Anopheles moucheti* [138, 141]  *Anopheles nevai* [139]  *Anopheles nili* [138, 141]  *Anopheles nuneztovari* [139, 143]  *Anopheles oswaldoi* [139]  *Anopheles ovengensis* [141]  *Anopheles paludis* [141]  *Anopheles pharoensis* [141]  *Anopheles pulcherrimus* [142]  *Anopheles quadrimaculatus* [145]  *Anopheles sacharovi* [138, 140]  *Anopheles sergentii* [138, 140]  *Anopheles stephensi* [146]  *Anopheles superpictus* [135]  *Anopheles triannulatus* [139, 143]  *Anopheles wellcomei* [141]  *Anopheles ziemanni* [141]  *Culex quinquefasciatus* [144]  *Culex stigmatosoma* [144]  *Culex tarsalis* [144] |
| Marituba (MTBV) | Family Bunyaviridae  Genus Bunyavirus |  | *Culex portesi* [1] | *Aedes aegypti* [1]  *Culex quinquefasciatus* [1] |  | *Culex aikenii* [1]  *Culex portesi* [1] |
| Mayaro (MAYV) | Family Togaviridae  Genus Alphavirus | *Haemagogus janthinomys* [147] | *Aedes* aegypti [148]  *Aedes albopictus* [149]  *Aedes scapularis* [105]  *Anopheles freeborni* [150]  *Anopheles gambiae* [150]  *Anopheles quadrimaculatus* [150]  *Anopheles stephansi* [150] | *Aedes aegypti* [148]  *Aedes albopictus* [149]  *Anopheles freeborni* [150]  *Anopheles gambiae* [150]  *Anopheles quadrimaculatus* [150]  *Anopheles stephansi* [150] | *Aedes aegypti* [148]  *Aedes albopictus* [149, 151]  *Anopheles freeborni* [150]  *Anopheles gambiae* [150]  *Anopheles quadrimaculatus* [150]  *Anopheles stephensi* [150] | *Aedes serratus* [152]  *Aedes aegypti* [151] |
| Mengovirus (MV) | Family PicornaviridaeGenus Cardiovirus |  |  |  |  | *Taeniorhynchus africanus* [153, 154]  *Taeniorhynchus fuscopennatus* [153, 154]  *Taeniorhynchus uniformis* [153, 154] |
| Middelburg (MIDV) | Family Togaviridae  Genus Alphavirus |  |  |  |  | *Mansonia africana* [18]  *Aedes caballus* [155]  *Aedes circumluteolus* [156]  *Aedes dalzieli* [157]  *Aedes lineatopennis* [1]  *Aedes palpalis* [1] |
| Murray Valley Encephalitis (MVEV) | Family Bunyaviridae  Genus Bunyavirus | *Culex annulirostris* [102] | *Culex quinquefasciatus* [158]  *Culex taeniopus* [49] | *Aedes aegypti* [49] |  | *Aedes serratus* [86, 159]  *Culex annulirostris* [160]  *Psorophora ferox* [88, 159] |
| Murutucu Virus (MURV) | Family Bunyaviridae  Genus Bunyavirus |  |  | *Aedes aegypti* [49] |  | *Culex aikenii* [161]  *Culex occosa* [83]  *Culex portesi* [161]  *Culex vomerifer* [83] |
| Negishi (NEGV) | Family Flaviviridae  Genus Flavivirus |  |  |  |  | *Aedes vexans* [162] |
| Nepuyo Virus (NEPV) | Family Bunyaviridae  Genus Bunyavirus |  | *Culex* sp. [163] |  | *Aedes aegypti* [164]  *Culex quinquefasciatus* [164] | *Culex accelerans* [165, 166]  *Culex* sp. [163]  *Culex taeniopus* [167, 168]  *Culex iolambdis* [169] |
| Nyando (NDOV) | Family Bunyaviridae  Genus Bunyavirus-like |  |  |  |  | *Aedes dalzieli* [1]  *Anopheles funestus* [170]  *Anopheles gambiae* [170]  *Eretmapodites* sp*.* [171] |
| Ockelbo (OCKV) | Family Togaviridae  Genus Alphavirus |  | *Aedes aegypti* [172]  *Aedes taeniorhynchus* [172]  *Culex pipiens* [173]  *Culex torrentium* [173] | *Aedes aegypti* [172]  *Aedes cinereus* [174]  *Aedes excrucians* [174]  *Aedes taeniorhynchus* [172]  *Culex pipiens* [173]  *Culex torrentium* [173] | *Aedes communis* [174] | *Aedes cinereus* [175]  *Culex pipiens* [175]  *Culex torrentium* [175]  *Culiseta morsitans* [175] |
| O'nyong'nyong (ONNV) | Family Togaviridae  Genus Alphavirus | *Anopheles funestus* [176]  *Anopheles gambiae* [176] |  |  |  | *Anopheles funestus* [170, 177, 178]  *Anopheles gambiae* [170, 177, 178]  *Mansonia uniformis* [1] |
| Oriboca (ORIV) | Family Bunyaviridae  Genus Bunyavirus |  | *Aedes aegypti* [1]  *Culex portesi* [96] |  |  | *Aedes taeniorhynchus* [1]  *Culex portesi* [46] |
| Oropouche (OROV) | Family Bunyaviridae  Genus Bunyavirus |  |  |  | *Aedes albopictus* [179] | *Aedes serratus* [180]  *Culex quinquefasciatus* [1]  *Mansonia venezuelensis* [181] |
| Orungo (ORUV) | Family Reoviridae  Genus Orbivirus |  |  | *Aedes albopictus* [182] | *Aedes aegypti* [182, 183] | *Aedes dentatus* [184]  *Anopheles funestus* [185]  *Anopheles gambiae* [186]  *Culex perfuscus* [187]  *Aedes taylori* [188] |
| Ossa (OSSAV) | Family Bunyaviridae  Genus Bunyavirus |  | *Culex taenipous* [1]  *Culex vomerifer*[1] |  |  |  |
| Pongola (PGAV) | Family Bunyaviridae  Genus Bunyavirus |  | *Aedes circumluteolus* [189] |  |  | *Aedes mcintoshi* [18]  *Aedes circumluteolus* [155]  *Aedes dalzieli* [157]  *Aedes tarsalis* [190]  *Aedes vittatus* [157]  *Anopheles coustani* [3]  *Anopheles funestus* [190]  *Mansonia africana* [189]  *Mansonia uniformis* [189] |
| Powassan (POW) | Family Flaviviridae  Genus Flavivirus |  |  |  |  | *Aedes togoi* [191]  *Anopheles hyrcanus* [191] |
| Restan (RESV) | Family Bunyaviridae  Genus Bunyavirus |  | *Aedes aegypti* [192] |  |  | *Culex portesi* [192] |
| Rift Valley (RFV) | Family Bunyavirus  Genus Phelbovirus |  | *Aedes aegypti* [1]  *Aedes albopictus* [193]  *Aedes argenteopunctatus* [193]  *Aedes atlanticus* [193]  *Aedes calceatus* [193]  *Aedes caballus* [194]  *Aedes canadensis* [193]  *Aedes canator* [193]  *Aedes circumluteolus* [193]  *Aedes dentatus* [193, 195]  *Aedes dorsalis* [193]  *Aedes excrucians* [193]  *Aedes fitchii* [193]  *Aedes fowleri* [193]  *Aedes implicatus* [193]  *Aedes infirmatus* [193]  *Aedes japonicus* [193]  *Aedes mcintoshi* [193, 196]  *Aedes notoscriptus* [193]  *Aedes ochraceus* [196]  *Aedes palpalis* [193]  *Aedes sollicitans* [193]  *Aedes sticticus* [193]  *Aedes taeniorhychus* [193]  *Aedes triseriatus* [193]  *Aedes unidentatus* [193]  *Aedes vexans* [193, 196]  *Aedes vigilax* [193]  *Anopheles crucians* [193]  *Anopheles multicolor* [193]  *Anopheles pharoensis* [193]  *Anopheles quadrimaculatus* [193]  *Coquillettidia perturbans* [193]  *Culex annulirostris* [193]  *Culex antennatus* [193]  *Culex erraticus* [193]  *Culex neavei* [193]  *Culex nigripalpus* [193]  *Culex perexiguus* [193]  *Culex pipiens* [193]  *Culex poicilipes* [193]  *Culex quinquefasciatus* [193]  *Culex restuans* [193]  *Culex rubinotus* [193]  *Culex salinarius* [193]  *Culex tarsalis* [193]  *Culex territam* [193]  *Culex theileri* [193]  *Culex tritaeniorhychus* [197]  *Culex univttatus* [193]  *Culieta minnesotae* [193]  *Culiseta inornata* [193]  *Eretmapodites quinquevittatus* [193]  *Mansonia dyari* [193]  *Psorophora ferox* [193] | *Aedes vexans* [197]  *Culex quinquefasciatus* [198]  *Culex zombaensis* [198] | *Aedes albopictus* [193]  *Aedes atlanticus* [193]  *Aedes calceatus* [193]  *Aedes canadensis* [193]  *Aedes canator* [193]  *Aedes circumluteolus* [193]  *Aedes dentatus* [193]  *Aedes dorsalis* [193]  *Aedes excrucians* [193]  *Aedes fitchii* [193]  *Aedes fowleri* [193]  *Aedes implicatus* [193]  *Aedes infirmatus* [193]  *Aedes juppi* [199]  *Aedes mcintoshi* [193]  *Aedes palpalis* [193]  *Aedes sollicitans* [193]  *Aedes sticticus* [193]  *Aedes taeniorhychus* [193]  *Aedes triseriatus* [193]  *Aedes unidentatus* [193]  *Aedes vexans* [193]  *Anopheles crucians* [193]  *Anopheles multicolor* [193]  *Coquillettidia perturbans* [193]  *Culex annulirostris* [193]  *Culex antennatus* [193]  *Culex erraticus* [193]  *Culex neavei* [193]  *Culex nigripalpus* [193]  *Culex perexiguus* [193]  *Culex pipiens* [193]  *Culex poicilipes* [193]  *Culex quinquefasciatus* [193, 198]  *Culex restuans* [193]  *Culex salinarius* [193]  *Culex tarsalis* [193]  *Culex territam* [193]  *Culex theileri* [193]  *Culex univttatus* [193]  *Culex zombaensis* [198, 199]  *Culiseta inornata* [193]  *Eretmapodites quinquevittatus* [193]  *Mansonia dyari* [193]  *Psorophora ferox* [193]  *Aedes detritus* [193]  *Aedes stimulans* [193]  *Aedes caballus* [193]  *Culex erythrothorax* [193] | *Aedes africanus* [196]  *Aedes caballus* [196, 197]  *Aedes caspius* [19]  *Aedes circumluteolus* [19, 196]  *Aedes dalzieli* [196]  *Aedes dendrophilus* [196]  *Aedes dentatus* [196]  *Aedes juppi* [199]  *Aedes mcintoshi* [196]  *Aedes ochraceus* [196]  *Aedes palpalis* [196]  *Aedes pembaensis* [19]  *Aedes tarsalis* [196]  *Aedes vexans* [196, 200]  *Anopheles pharoensis* [196]  *Anopheles coustani* [193]  *Anopheles arabiensis* [193]  *Anopheles cincereus* [193]  *Anopheles squamosus* [18]  *Coquillettidia fuscopennata* [193]  *Culex bitaeniorhychus* [18]  *Culex antennatus* [193]  *Culex neavei* [193]  *Culex perexiguus* [197]  *Culex pipiens* [193]  *Culex poicilipes* [18, 193]  *Culex quinquefasciatus* [193]  *Culex theileri* [193]  *Culex tritaeniorhychus* [197]  *Culex univttatus* [193]  *Culex zombaensis* [19, 199]  *Eretmapodites chrysogaster* [1]  *Eretmapodites quinquevittatus* [18]  *Mansonia africana* [18]  *Mansonia uniformis* [18] |
| Rocio (ROCV) | Family Flaviviridae Genus Flavivirus |  | *Aedes scapularis* [201]  *Culex nigripalpus* [202]  *Culex opisthopus* [202]  *Culex pipiens* [202]  *Culex tarsalis* [202]  *Psorophora ferox* [1, 202]  *Culex quinquefasciatus* [202] |  | *Aedes serratus* [203]  *Culex quinquefasciatus*  [202] | *Aedes scapularis* [201]  *Coquillettidia chrysonotum* [201]  *Mansonia indubitans* [201]  *Psorophora ferox* [1, 202] |
| Ross River (RRV) | Family Togaviridae  Genus Alphavirus | *Aedes camptorynchus* [204]  *Aedes notoscriptus* [204]  *Aedes vigilax* [204]  *Culex annulirostris* [204] | *Aedes albopictus* [205]  *Aedes notoscriptus* [206] | *Aedes albopictus* [64]  *Aedes camptorynchus* [207]  *Aedes notoscriptus* [206]  *Coquillettidia linealis* [208]  *Ochlerotatus vigilax* [208] | *Aedes albopictus*  [205]  *Aedes funereus* [209]  *Aedes multiplex* [209]  *Aedes notoscriptus* [209]  *Aedes procax* [209]  *Aedes vigilax* [209]  *Culex annulirostris* [209]  *Culex australicus* [209]  *Mansonia uniformis* [209] | *Aedes alternans* [210]  *Aedes funereus* [210]  *Aedes notoscriptus* [210]  *Aedes procax* [210]  *Aedes vigilax* [210]  *Anopheles amictus* [211]  *Coquillettidia linealis* [211]  *Culex annulirostris* [210]  *Culex antennatus* [212]  *Culex sitiens* [210] |
| Semliki Forest (SFV) | Family Togaviridae  Genus Alphavirus |  | *Aedes aegypti* [1]  *Aedes togoi* [1]  *Anopheles albimanus* [1]  *Anopheles quadrimaculatus* [1] |  | *Culex salinarius* [1]  *Culex annulirostris* [213] | *Aedes aegypti* [214]  *Aedes abnormalis* [215]  *Aedes africanus* [214]  *Aedes argenteopunctatus* [216]  *Aedes mcintoshi* [17]  *Aedes ochraceus* [17]  *Aedes opok* [214]  *Aedes palpalis* [214]  *Aedes vexans* [217]  *Aedes vittatus* [214]  *Culex pipiens* [217]  *Culex quinquefasciatus* [214]  *Eretmapodites chrysogaster* [214]  *Eretmapoites* sp [218] |
| Sepik (SEPV) | Family Flaviviridae Genus Flavivirus |  |  |  |  | *Armigeres* sp*.* [1]  *Culex sitiens* [216]  *Ficalbia flavens* [1]  *Ficalbia* sp*.* [217]  *Mansonia septempunctata* [217] |
| Shokwe (SHOV) | Family Bunyavirus  Genus  Orthobunyavirus |  |  |  |  | *Aedes argenteopunctatus* [157]  *Aedes circumluteolus* [1]  *Aedes cumminsii* [1]  *Aedes dalzieli* [157]  *Aedes dentatus* [221]  *Anopheles brohieri* [221]  *Mansonia Africana* [1] |
| Sindbis (SINV) | Family Togaviridae  Genus Alphavirus |  | *Culex torrentium* [222]  *Aedes aegypti* [223]  *Culex pipiens* [224]  *Culex univittatus* [225] | *Culex torrentium* [222]  *Culex neavei* [226]  *Culex pipiens* [224]  *Culex univittatus* [225] | *Culex torrentium* [222]  *Culex theileri* [87]  *Culex univittatus* [225] | *Aedes mormanensis* [227]  *Aedes* [227]  *Anopheles maculipennis* [228]  *Anopheles pharoensis* [229]  *Culex annulirostris* [230]  *Culex atennatus* [229]  *Culex bitaeniorhynchus* [230]  *Culex neavei* [226]  *Culex pseudovishnui* [231]  *Culex quinquefasciatus* [227]  *Culex theileri* [87]  *Culex tritaeniorhynchus* [1]  *Mansonia fuscopennata* [232]  *Mansonia semptempunctata* [227] |
| Snowshoe hare (SSH) | Family Bunyaviridae Genus Bunyavirus |  | *Aedes aegypti* [38]  *Aedes cinereus* [233]  *Culiseta inornata* [38]  *Aedes provocans* [115]  *Aedes triseriatus* [234] |  |  | *Aedes canadensis* [233]  *Aedes cataphylla* [233]  *Aedes cinereus* [233]  *Aedes communis* [233]  *Aedes excrucians* [233]  *Aedes fitchii* [235]  *Aedes hexodontus* [233]  *Aedes punctor* [233]  *Aedes stimulans* [233]  *Culiseta impatiens* [236]  *Culiseta inornata* [233] |
| Spondweni (SPONV) | Family Flaviviridae  Genus Flavivirus | *Aedes circumluteolus* [216]  *Mansonia africana* [216]  *Mansonia uniformis* [155] | *Aedes aegypti* [237]  *Aedes circumluteolus* [216] | *Aedes aegypti* [237]  *Aedes albopictus* [238]  *Culex quinquefasciatus* [238] |  | *Aedes circumluteolus* [216]  *Aedes cumminsii* [216]  *Aedes fryeri/fowleri* [239]  *Culex neavei* [156]  *Culex univitattus* [18]  *Eretmapodites silvestris* [216]  *Eretmapodites* sp*.* [240]  *Mansonia africana* [216]  *Mansonia uniformis* [155] |
| St. Louis Encephalitis (SLE) | Family Flaviviridae  Genus Flavivirus | *Culex declarator* [73]  *Culex nigripalpus* [241]  *Culex pipiens* [241]  *Culex quinquefasciatus* [241]  *Culex tarsalis* [241] | *Aedes albopictus* [242]  *Aedes japonicus* [243] *Aedes lateralis* [135, 244]  *Aedes negromaculis* [135, 244]  *Aedes vexans* [135, 244]  *Culex coronator* [135, 244]  *Culex pipiens* [135, 244]  *Culex quinquefasciatus* [245]  *Culex restuans* [246]  *Culex salinarius* [246]  *Culex stigmatosoma* [245]  *Culex tarsalis* [135, 244, 245]  *Theobaldi incidens* [135, 244]  *Theobaldia inornata* [135, 244] |  | *Aedes aegypti* [246]  *Aedes albopictus* [247]  *Aedes atropalpus* [248]  *Aedes dorsalis* [249]  *Aedes epaticus* [247]  *Aedes japonicus* [243]  *Aedes lateralis* [135, 244]  *Aedes negromaculis* [135, 244]  *Aedes vexans* [135, 244]  *Anopheles maculipennis* [135, 244]  *Culex coronator* [135, 244]  *Culex peus* [249]  *Culex pipiens* [135, 244]  *Culex quinquefasciatus* [135, 244, 245]  *Culex restuans* [246]  *Culex salinarius* [246]  *Culex stigmatosoma* [135, 244, 245]  *Culex tarsalis* [135, 244, 245]  *Mansonia perterbons* [246]  *Psorophora ciliata* [135, 244]  *Theobaldi incidens* [135, 244]  *Theobaldia inornata* [135, 244]  *Wyeomyia vanduzeei* [135, 244] | *Aedes serratus* [180]  *Aedes taeniorhynchus* [250]  *Anopheles crucians* [250, 251]  *Anopheles quadrimaculatus* [252]  *Culex coronator* [73, 135, 244]  *Culex tarsalis* [253]  *Mansonia titillans* [254] |
| Tacaiuma (TCMV) | Family Bunyaviridae  Genus Bunyavirus |  |  |  | *Aedes aegypti* [1]  *Anopheles quadrimaculatus* [1] | *Anopheles cruzii* [1]  *Haemagogus* sp*.* [1] |
| Tahyna (TAHV) | Family Bunyaviridae  Genus Bunyavirus | *Culex pipiens* [255, 256] | *Aedes caspius* [257]  *Aedes sticticus* [258]  *Aedes vexans* [258, 259]  *Culiseta annulata* [258] | *Aedes caspius* [257]  *Aedes communis* [1]  *Aedes sticticus* [1]  *Aedes vexans* [259]  *Culiseta annulata* [1] | *Aedes caspius* [257]  *Aedes vexans* [259] | *Aedes albopictus* [260]  *Aedes detritus* [91, 260]  *Aedes cantans* [2, 258, 261]  *Aedes caspius* [258, 259]  *Aedes cinereus* [259, 260]  *Aedes communis* [262]  *Aedes diantaeus* [9, 258]  *Aedes pembaensis* [258]  *Aedes sticticus* [255, 256, 258, 263]  *Aedes vexans* [255, 258, 259]  *Anopheles hyrcanus* [1, 264]  *Coquillettidia richiardii* [255, 256]  *Culex modestus* [258, 265]  *Culex pipiens* [255, 256]  *Culiseta annulata* [258, 264] |
| Tataguine (TATV) | Family Bunyaviridae  Genus Bunyavirus-like |  |  |  |  | *Anopheles funestus* [1]  *Anopheles gambiae* [1]  *Mansonia aurites* [1] |
| Tensaw (TSV) | Family Bunyaviridae  Genus Bunyavirus |  | *Anopheles* sp*.* [266] |  |  | *Aedes taeniorhynchus* [267]  *Aedes atlanticus* [267]  *Aedes infirmatus* [267]  *Aedes mitchellae* [267]  *Anopheles crucians* [267]  *Anopheles punctipennis* [267]  *Anopheles quadrimaculatus* [267]  *Culex nigripalpus* [267]  *Culex salinarius* [267]  *Mansonia perturbans* [267]  *Psorophora confinnis* [267] |
| Tonate (TONV) | Family Togaviridae  Genus Alphavirus |  |  |  |  | *Anopheles brasiliensis* [1]  *Anopheles mediopunctatus* [1]  *Coquillettidia albicosta* [1]  *Coquillettidia venezuelensis* [1]  *Culex portesi* [1]  *Culex spissipes* [1]  *Culex zeteki* [1]  *Culex taeniopus* [268]  *Mansonia pseudotitillans* [1]  *Mansonia titillans* [1]  *Wyeomyia melanocephala* [1]  *Wyeomyia occulta* [1]  *Wyeomyia pseudopecten* [1] |
| Trivittatus (TVTV) | Family Bunyaviridae  Genus Bunyavirus | *Aedes trivittatus* [269] |  |  |  | *Aedes atlanticus* [1]  *Aedes infirmatus* [270]  *Aedes sticticus* [271]  *Aedes taeniorhynchus* [1]  *Aedes trivittatus* [269, 271]  *Aedes vexans* [1, 271]  *Culex pipiens* [1]  *Mansonia perturbans* [1]  *Culex tarsalis* [271] |
| Tularemia | Family Francisellacea  Genus Francisella |  | *Aedes aegypti* [272, 273]  *Aedes vexans* [135, 274] |  |  | *Aedes cinereus* [135, 273, 274]  *Aedes communis* [273]  *Aedes punctor* [273]  *Aedes sticticus* [273]  *Aedes vexans* [273]  *Anopheles claviger* [275]  *Anopheles maculipennis* [275]  *Culex modestus* [275]  *Ochlerotatus excrucians* [135, 274] |
| Usutu (USUV) | Family Flaviviridae  Genus Flavivirus | *Culex neavei* [276]  *Culex pipiens* [277] |  |  |  | *Aedes albopictus* [277]  *Aedes capoius* [277]  *Anopheles maculipennis* [277]  *Culex atennatus* [278]  *Culex univittatus* [18]  *Culex neavei* [279]  *Culex perfuscus* [221]  *Culex perexigus* [280]  *Culex pipiens* [277, 281, 282]  *Culex torrentium* [283]  *Coquillettidia aurites* [157]  *Mansonia africana* [157] |
| Venezuelan Equine Encephalitis (VEE) | Family Togaviridae  Genus Alphavirus | *Culex taeniopus* [284] | *Aedes aegypti* [1]  *Aedes albopictus* [285]  *Aedes fulvus* [286]  *Aedes mediovittatus* [1]  *Aedes sollicitans* [1]  *Aedes taeniorhynchus* [285]  *Aedes triseriatus* [1]  *Aedes vexans* [1]  *Aedess atropalpus* [1]  *Anopheles freeborni* [1]  *Anopheles quadrimaculatus* [1]  *Anopheles stephensi* [1]  *Culex taeniorhynchus* [1]  *Culex vomerifer* [286]  *Culex quinquefasciatus* [1]  *Culex tarsalis* [1]  *Mansionia indubitans* [286]  *Psorophora cingulata*[286]  *Psorophora confinnis* [1] | *Aedes albopictus* [285]  *Aedes fulvus* [286]  *Aedes serratus*[286]  *Aedes taeniorhynchus* [285]  *Culex coronator* [286]  *Culex vomerifer* [286]  *Mansionia indubitans* [286]  *Psorophora cingulata*[286] | *Aedes taeniorhynchus* [287] | *Aedes aegypti* [1]  *Aedes mediovittatus* [1]  *Aedes scapularis* [1]  *Aedes sollicitans* [1]  *Aedes taeniorhynchus* [288]  *Aedes thelcter* [1]  *Anopheles aquasalis* [288]  *Anopheles crucians* [1]  *Anopheles neomaculipalpus* [1]  *Anopheles pesudopenctipennis* [1]  *Anopheles punctimacula* [1]  *Culex corniger* [1]  *Culex ocossa* [1]  *Culex quinquefasciatus* [1]  *Culex tarsalis* [1]  *Deinocerites pseudes* [1]  *Mansonia dyari* [1]  *Psorophora ciliata* [1]  *Psorophora confinnis* [288]  *Psorophora cyanescens* [1]  *Psorophora discolor* [1] |
| Wanowrie (WAN) | Unassigned |  |  |  |  | *Culex quinquefasciatus* [1, 289] |
| West Nile Virus (WNV) | Family Flaviviridae  Genus Flavivirus | *Culex modestus* [290]  *Culex pipiens* [286]  *Culex quinquefasciatus* [286]  *Culex tarsalis* [291] | *Aedes aegypti* [248, 292]  *Aedes albopictus* [135, 248, 293, 294]  *Aedes atropalpus* [248]  *Aedes caspius* [292]  *Aedes dorsalis* [295]  *Aedes japonicus* [248, 296]  *Aedes melanimon* [295]  *Aedes sollicitans* [248]  *Aedes sierrensis* [295]  *Aedes taeniorhynchus* [248]  *Aedes vexans* [286, 295]  *Coquillettidia perturbans* [296]  *Culex antennatus* [292]  *Culex erythrothorax* [295]  *Culex fusocephala* [293]  *Culex nigripalpus* [296]  *Culex pipiens* [248, 286, 292, 295, 296, 297]  *Culex quinquefasciatus* [292, 293, 295, 296]  *Culex restuans* [296]  *Culex salinarius* [296]  *Culex stigmatosoma* [295, 296]  *Culex tarsalis* [295, 296]  *Culex theileri* [292]  *Culex tritaeniorhynchus* [292, 293]  *Culex univittatus* [292]  *Culex vishnui* [292, 293]  *Culiseta inornata* [295] | *Aedes sollicitans* [286]  *Aedes vexans* [286]  *Culex pipiens* [286] | *Aedes dorsalis* [295]  *Aedes melanimon* [295]  *Aedes sierrensis* [295]  *Aedes sollicitans* [286]  *Aedes vexans* [286, 295]  *Coquillettidia perturbans* [296]  *Culex erythrothorax* [295]  *Culex nigripalpus* [296]  *Culex pipiens* [83, 286, 295]  *Culex quinquefasciatus* [295, 296]  *Culex restuans* [296]  *Culex salinarius* [296]  *Culex stigmatosoma* [295]  *Culex tarsalis* [295]  *Culiseta inornata* [295] | *Aedes dalzieli* [1]  *Anopheles rufipes* [1]  *Aedes aegypti* [83, 292]  *Aedes albopictus* [83]  *Aedes atropalpus* [83]  *Aedes canadensis* [298]  *Aedes cantans* [275]  *Aedes caspius* [292]  *Aedes japonicus* [83, 299]  *Aedes sollicitans* [83]  *Aedes taeniorhynchus* [83]  *Aedes triseriatus* [298, 299]  *Aedes vexans* [292, 298, 299, 300]  *Anopheles brunnipes* [292]  *Anopheles coustani* [301]  *Anopheles maculipalpis* [292]  *Anopheles maculipennis* [275, 292]  *Anopheles plumbeus* [275]  *Anopheles punctipennis* [299]  *Anopheles subpictus* [292]  *Coquillettidia metallica* [292]  *Coquillettidia microannulata* [292]  *Coquillettidia richiardii* [292]  *Culex antennatus* [229, 292]  *Culex decens* [292]  *Culex ethiopicus* [292]  *Culex guiarti* [292]  *Culex modestus* [292]  *Culex neavei* [292]  *Culex nigripes* [292]  *Culex perexigus* [292]  *Culex perfuscus* [292]  *Culex pipiens* [292, 298, 299, 300]  *Culex poicilipes* [292]  *Culex pruina* [292]  *Culex quinquefasciatus* [292]  *Culex restuans* [298, 299]  *Culex salinarius* [298, 299]  *Culex scottii* [292]  *Culex theileri* [87, 292]  *Culex tritaeniorhynchus* [292]  *Culex univittatus* [83, 87, 229, 292, 302]  *Culex vishnui* [292]  *Culex weschei* [292]  *Culisetsa melanura* [298]  *Mansonia uniformis* [292]  *Mimomyia hispida* [292]  *Mimomyia lacustris* [292]  *Mimomyia splendens* [292]  *Ochlerotatus geniculatus* [275]  *Orthopodymia signifera* [302] |
| Western Equine Encephalitis (WEE) | Family Togaviridae  Genus Alphavirus | *Aedes melanimon* [303]  *Culex tarsalis* [304] | *Aedes aegypti* [305]  *Aedes albopictus* [305]  *Culex quinquefasciatus* [305]  *Culex pipiens pallens* [305]  *Culex tritaeniorhynchus* [305] | *Aedes aegypti* [305]  *Aedes albopictus* [305]  *Culex. quinquefasciatus* [305]  *Culex pipiens pallens* [305]  *Culex tritaeniorhynchus* [305] | *Aedes aegypti* [305]  *Aedes albopictus* [305]  *Culex quinquefasciatus* [305]  *Culex pipiens pallens* [305]  *Culex tritaeniorhynchus* [305] | *Aedes albifasciatus* [64]  *Anopheles albitarsis* [64]  *Mansonia* sp*.* [64]  *Psorophora pallescens* [64] |
| Whataroa (WHAV) | Family Togaviridae  Genus Alphavirus |  | *Aedes australis* [306]  *Culiseta tonnoiri* [306] |  |  | *Culex pervigilans* [307]  *Culiseta tonnoiri* [306] |
| Witwatersrand (WITV) | Family Bunyaviridae  Genus Bunyavirus-like |  | *Culex rubinotus* [87] |  |  | *Culex rubinotus* [308]  *Aedes argyrothorax* [1] |
| Wyeomyia Virus (WYOV) | Family Bunyaviridae  Genus Bunyavirus |  |  |  |  | *Aedes argyrothorax* [1]  *Aedes fulvus* [309]  *Aedes scapularis* [310]  *Aedes septemstriatus* [24]  *Aedes septemstriatus* [24]  *Aedes serratus* [24]  *Aedes sexlineatus* [24]  *Aedes sexlineatus* [24]  *Aediomyia squamipennis* [83]  *Anopheles nimbus* [1]  *Coquillettidia arribalzagai* [1]  *Culex amazonesis* [310]  *Culex nigripalpus* [23]  *Hemagogus leucocephalus* [1]  *Limatus asulleptus* [22]  *Limatus flaisetosus* [83]  *Ochlerotatus fulvus* [83]  *Psorophora albigenu* [22, 83]  *Psorophora cingulata* [1]  *Psorophora ferox* [22, 83]  *Trichoprosopon digitatum* [46]  *Trichoprosopon leucopus* [1]  *Trichoprosopon longipes* [1]  *Wyeomyia aporonoma* [1]  *Wyeomyia complosa* [1]  *Wyeomyia melanocephala* [58, 98, 311]  *Wyeomyia occulta* [59]  *Wyeomyia* sp*.* [46] |
| Yellow Fever (Jungle) (YFVJ) | Family Flaviviridae  Genus Flavivirus | *Aedes aegypti* [316] | *Aedes fluviatilis* [317, 318]  *Aedes scapularis* [318, 319]  *Aedes taeniorhynchus* [317, 320]  *Haemagogus janthinomys* [321] |  |  |  |
| Yellow Fever (Urban) (YFVU) | Family Flaviviridae  Genus Flavivirus | *Aedes aegypti* [318] | *Aedes vittatus* [319]  *Eretmapodites quinquevittatus* [320] |  | *Aedes aegypti* [64, 318]  *Aedes albopictus* [64, 321]  *Aedes apicoannulatus* [322]  *Aedes luteocephalus* [322] | *Aedes africanus* [323]  *Aedes metallicus* [18]  *Aedes opok* [18]  *Aedes serratus* [324]  *Aedes simpsoni* [323]  *Aedes vittatus* [18]  *Anopheles nevai* [325]  *Hemagogus equinus* [325]  *Hemagogus leucocephalus* [325]  *Hemagogus lucifer* [325]  *Hemagogus spegazzinii* [18, 325]  *Hememagogus capricorni* [18]  *Hememagogus mesodentatus* [18]  *Sebethes chloropterus* [325] |
| Zika (ZIKV) | Family Flaviviridae  Genus Flavivirus | *Aedes aegypti* [326, 327, 328, 329]  *Aedes albopictus* [326, 328, 329] | *Aedes aegypti* [328, 330]  *Aedes luteocephalus* [53, 327]  *Aedes vittatus* [53, 327] | *Aedes aegypti* [326, 327, 328, 330]  *Aedes africanus* [327, 328]  *Aedes albopictus* [326, 328]  *Aedes hensilli* [50, 328]  *Aedes luteocephalus* [53, 327]  *Aedes polynesiensis* [330]  *Aedes vexans* [328]  *Aedes vittatus* [53, 327] | *Aedes aegypti* [326, 330]  *Aedes albopictus* [326]  *Aedes hensilli* [50, 328]  *Aedes luteocephalus* [53, 327]  *Aedes polynesiensis* [330]  *Aedes triseriatus* [326]  *Aedes unilineatus*  [53]  *Aedes vexans* [328]  *Aedes vittatus* [53, 327]  *Culex quinquefasciatus* [328] | *Aedes aegypti* [327]  *Aedes taeniorhynchus* [331]  *Aedes africanus* [53, 327, 329]  *Aedes apicoargenteus* [53]  *Aedes dalzieli* [329, 332]  *Aedes furcifer* [53, 327, 329]  *Aedes hirsutus* [327, 329]  *Aedes luteocephalus* [53, 329]  *Aedes metallicus* [327, 328, 329]  *Aedes opok* [327, 328]  *Aedes taylori* [53, 329]  *Aedes unilineatus* [327, 329]  *Aedes vittatus* [53, 329]  *Anopheles coustani* [4, 5]  *Anopheles gambiae* [53]  *Culex perfuscus* [327, 328, 329]  *Mansonia uniformis* [328, 329]  *Armigeres subbalbeatus* [91] |

**Additional file 1: Table S1.** **Mosquito vectors associated with pathogens of human disease relevance**. For each pathogen/disease (with abbreviation), species of mosquito that fall into five categories are listed. Wild infection are those that have been found to carry the virus during sampling of mosquitoes collected in nature, Lab infection are those who were positive for a virus after offered an infectious blood meal, Lab Dissemination are those that showed replication of the virus in tissue (e.g., legs), Lab Transmit were those that could pass the pathogen on to a host under laboratory conditions (often to a non-human mammal), and Known Vectors were those that were considered to be a central species in maintaining the pathogen in nature and directly infecting humans. In all cases we assumed species names were used as *sensu stricto* (e.g., *Anopheles gambiae*) based on the publications that listed them. We cannot know for sure in all cases as many publications did not list s.s. or s.l., but given the nature of those publications we assemed they were s.s.

**Literature Cited**

1. Centers for Disease Control and Prevention (CDC). Arbovirus catalog. 1985.

2. Smithburn KC, Haddow AJ, Mahaffy AF. A neurotropic virus isolated from *Aedes* mosquitoes caught in the Semliki forest. Am J Trop Med. 1946;26:189-208.

3. Metselaar D, Henderson BE, Kirya GB, Tukei PM, de Geus A. Isolation of arboviruses in Kenya, 1966-1971. Trans R Soc Trop Med Hyg.1974;68:114-23.

4. Lvov DK, Karas FR, Tsyrkin YM, Vargina SG, Timofeev EM, Osipova NZ, et al. 1974. Batken virus, a new arbovirus isolated from ticks and mosquitoes in Kirghiz S.S.R. Arch Gesamte Virusforsch. 1974;44:70-73.

5. Nabeshima T, Nga PT, Guillermo P, del Carmen Parquet, M,Yu F, Thanh Thuy N, et al. Isolation and molecular characterization of Banna virus from mosquitoes, Vietnam. Emerg Infect Dis. 2008;14:1276-9.

6. Watson TM, Kay BH. Vector competence of *Aedes notoscriptus* (Diptera: Culicidae) for Barmah Forest virus and of *Aedes aegypti* (Diptera: Culicidae) for dengue 1-4 viruses in Queensland, Australia. J Med Entomol. 1999;36:508-514.

7. Ryan PA and Kay BH. Vector competence of mosquitoes (Diptera: Culicidae) from Maroochy Shire, Australia, for Barmah Forest virus. J Med Entomol. 1999;36:856-860.

8. Boyd AM, Kay BH. Experimental infection and transmission of Barmah Forest virus by *Aedes vigilax* (Diptera: Culicidae). J Med Entomol. 1999;36:186-189.

9. Smithburn KC, Haddow AJ, Mahaffy AF. A Neurotropic Virus Isolated from Aedes Mosquitoes Caught in the Semliki Forest. Am J Trop Med. 1946;26:189-208.

10. Centers for Disease Control and Prevention (CDC). Arbovirus catalog. 1985.

11. Collins O, Venter M, Chepkorir E, Mbaika A, Lutomiah J, Swanepoel R, Sang R; Vector competence of selected mosquito species in kenya for Ngari and Bunyamwera Viruses, J Med Entomol, 2014;51:1248–1253.

12. Whitman, L. Personal communication. Collected from: Bunyamwera Virus. Arbovirus Catalog. Centers for Disease Control and Prevention. <https://wwwn.cdc.gov/arbocat/VirusDetails.aspx?ID=79&SID=9>

13. Ajamma YU, Onchuru TO, Ouso DO, Omondi D, Masiga DK, Villinger J. Vertical transmission of naturally occurring Bunyamwera and insect-specific flavivirus infections in mosquitoes from islands and mainland shores of Lakes Victoria and Baringo in Kenya. PLoS Negl Trop Dis. 2018;12:e0006949.

14. Karabatsos N. International catalogue of arboviruses, including certain other viruses of vertebrates. Am Soc Trop Med. San Antonio, TX. 1985.

15. Kokernot RH, Smithburn KC, de Meillon B, Paterson HE. Isolation of Bunyamwera virus from a naturally infected human being and further isolations from *aedes* (Banksinelld) *circumluteolus* Theo. Am J Trop Med. 1958;7:579-84.

16. Ochieng C, Lutomiah J, Makio A, Koka H, Chepkorir E, Yalwala S, et al. Mosquito-borne arbovirus surveillance at selected sites in diverse ecological zones of Kenya; 2007–2012. Virology journal. 2013;10:1-0.

17. Crabtree M, Sang R, Lutomiah J, Richardson J, Miller B. Arbovirus surveillance of mosquitoes collected at sites of active Rift Valley fever virus transmission: Kenya, 2006-2007. J Med Entomol. 2009;46:961-964.

18. Braak L, Gouveia de Almeida AP, Cornel AJ, Swanepoel R, de Jager C. Mosquito-borne arboviruses of African origin: review of key viruses and vectors. Parasites Vectors. 2018;11:29.

19. Logan TM, Linthicum KJ, Davies FG, Binepal YS, Roberts CR. Isolation of Rift Valley fever virus from mosquitoes (Diptera: Culicidae) collected during an outbreak in domestic animals in kenya. J Med Entomol. 1991;28:293–295.

20. Tauro LB, Rivarola ME, Lucca E, Mariño B, Mazzin Ri, Ferreira Cardoso J, et al. First isolation of Bunyamwera virus (Bunyaviridae family) from horses with 5 neurological diseases and an abortion in Argentina. Vet J. 2015;206:111–114

21. Beranek MD, Gallardo R, Almiron WR, Contigiani MS. First detection of *Mansonia titillans* (Diptera: Culicidae) infected with St. Louis encephalitis virus (Flaviviridae: Flavivirus) and Bunyamwera serogroup (Peribunyaviridae: Orthobunyavirus) in Argentina. J Vect Ecol. 2018;43:340-343.

22. Mores CN, Turell MJ, Dyer J, Rossi CA. Phylogenetic relationships among Orthobunyaviruses isolated from mosquitoes captured in Peru. Vector Borne Zoonotic Dis. 2009;9:25-32.

23. Galindo P, Srihongse S, de Rodaniche E, Grayson MA. An ecological survey for arboviruses in Almirante, Panama, 1959-1962. Am J Trop Med. 1966;15:385-400.

24. Causey OR, Causey CE, Maroja OM, Macedo DG. The isolation of arthropod-borne viruses, including members of two hitherto undescribed serological groups, in the Amazon region of Brazil. Am J Trop Med. 1961;10; 227-49.

25. Gonzalez JP, Georges AJ. Buyamweral fevers: Bunyamwera, Ilesha, Germiston, Bwamba and Tataguine. In T.P. Monath (ed.). The arboviruses: epidemiology and ecology. Boca Raton (FL): CRC Press.1988;18:84-98.

26. Lutwama JJ, Rwaguma EB, Nawanga PL, Mukuye A. Isolations of Bwamba virus from south central Uganda and north eastern Tanzania. Afr Health Sci. 2002;2:24-28.

27. Yuill TM, Thompson PH. Cache Valley virus in the Del Mar Va Peninsula. IV. Biological transmission of the virus by *Aedes sollicitans* and *Aedes taeniorhynchus*. Am J Trop Med. 1970;19:513-519.

28. Blackmore CG, Blackmore MS, Grimstad PR. Role of *Anopheles quadrimaculatus* and *Coquillettidia perturbans* (Diptera: Culicidae) in the transmission cycle of Cache Valley virus (Bunyaviridae: Bunyavirus) in the midwest, USA. J Med Entomol. 1998;35:660-664.

29. Centers for Disease Control and Prevention. Cache Valley Virus. Arbovirus Catalog.

30. Mitchell CJ, Haramis LD, Karabatsos N, Smith GC, Starwalt VJ. Isolation of La Cross, Cache Valley, and Potosi viruses from *Aedes* mosquitoes (Diptera: Culicidae) collected at used-tire sites in Illinois during 1994-1995. J Med Entomol. 1998;35:573-577.

31. Armstrong PM, Andreadis TG, Anderson JF. Emergence of a new lineage of Cache Valley virus (Bunyaviridae: Orthobunyavirus) in the northeastern United States. Am J Trop Med. 2015;93:11-17.

32. Buescher EL, Byrne RJ, Clarke GC, Gould DJ, Russell PK, Scheider FG, et al. Cache Valley virus in the Del Mar Va Peninsula. I. Virologic and serologic evidence of infection. Am J Trop Med. 1970;19:493-502.

33. Belle EA, Grant LS, Griffiths BB. The isolation of Cache Valley virus from mosquitoes in Jamaica. J W Indian Med.1966;15:217–220.

34. Calisher CH, Francy DB, Smith GC, Muth DJ, Lazuick JS, Karabatsos N, et al. Distribution of Bunyamwera serogroup viruses in North America, 1956-1984. Am J Trop Med. 1986;35:429-443.

35. Kokernot RH, Hayes J, Tempelis CH, Chan DHM, Boyd, KR, Anderson RJ. Arbovirus studies in the Ohio-Mississippi Basin, 1964-1967 IV. Cache Valley Virus. Am J Trop Med. 1969;18:768-773.

36. Holden P, Hess AD. Cache Valley virus, a previously undescribed mosquito-borne agent. Science 1959;130:1187-8.

37. Eklund, C. Personal communication. Collected from: Cache Valley Virus. Arbovirus Catalog. Centers for Disease Control and Prevention.

38. Chamberlain, R.W. Personal communication. Collected from: Cache Valley Virus. Arbovirus Catalog. Centers for Disease Control and Prevention.

39. Burgdorfer W, Newhouse VF, Thomas LA. Isolation of California encephalitis virus from the blood of a snowshoe hare (*Lepus arnericanus*) in western Montana. Am J Hyg. 1961;73:344-349.

40. McLean DM, Clarke AM, Coleman JC, Montalbetti CA, Skidmore AG, Walters TE, et al. Vector capability of *Aedes aegypti* mosquitoes for California encephalitis and dengue viruses at various temperatures. Cad J Microbiol. 1974;20:255-262.

41. Hammon W, Reeves W. California encephalitis virus, a newly described agent. III. Mosquito infection and transmission. J Immunol. 1952;69:511-514.

42. Hammon WM, Reeves WC, Sather G. California encephalitis virus, a newly described agent. II. Isolations and attempts to identify and characterize the agent. J Immunol.1952;69:493-510.

43. Eldridge BF, Glaser C, Pedrin RE, Chiles RE. The first reported case of California encephalitis in more than 50 years. Emerg Infect Dis. 2001;7:451-452.

44. Sudia WD, Newhouse VF,Calisher CH, Chamberlain RW. California group arboviruses: isolations from mosquitoes in North America. Mosq News. 1971;31:576-600.

45. Metselaar D. Isolation of arboviruses of group A and group C in Surinam. Trop Geogr Med. 1966;18:137-42.

46. Auguste AJ, Adams AP, Arrigo NC, Martinez R, da Rosa APT, Adesiyun AA, et al. Isolation and characterization of sylvatic mosquito-borne viruses in Trinidad: enzootic transmission and a new potential vector of Mucambo virus. Am J Trop Med. 2010;83: 1262-5.

47. Tsetsarkin KA, Vanlandingham DI, McGee CE, Higgs S. A single mutation in Chikungunya virus affects vector specificity and endemic potential. PLoS Pathog. 2007;3:e201.

48. Näslund J, Ahlm C, Islam K, Evander M, Bucht G, Wesula Lwande O. Emerging mosquito-borne viruses linked to *Aedes aegypti* and *Aedes albopictus*: Global status and preventive strategies. Vector Borne Zoonotic Dis. 2021.731-746.

49. Theiler M, Downs WG. The arthropod-borne viruses of vertebrates: An account of the Rockefeller Foundation Virus Program, 1951-1970. Yale University Press, New Haven, CT. 1973.

50. van den Hurk AF, Hall-Mendelin S, Pyke AT, Smith GA, Mackenzi JS. Vector competence of Australian mosquitoes for Chikungunya virus. Vector-Borne and Zoonotic Dis. 2010;10:489-495.

51. Shak KV, Gilotra, SK, Gibbs CJ, Rozeboom LE. Laboratory studies of transmission of chikungunya virus by mosquitoes: A preliminary report. Indian J. Med. Res. 1964; 52:703-709.

52. Ledermann JP, Guillaumot L, Yug L, Saweyog SC, Tided M, Machieng P, et al. *Aedes hensilli* as a potential vector of Chikungunya and Zika viruses. PLoS Neg Trop Dis. 2014;8: e3188.

53. Diagne CT, Diallo D, Faye O, Ba Y, Faye O, Gaye A, et al. Potential of selected Senegalese *Aedes* spp. mosquitoes (Diptera: Culicidae) to transmit Zika virus. BMC Infect Dis. 2015;15: 492.

54. Talbalaghi A, Moutailler S, Vazeille M, Failloux AB. Are *Aedes albopictus* or other mosquito species from northern Italy competent to sustain new arboviral outbreaks?. Med Vet Entomol 2010;24:83-7.

55. Coffey LL, Failloux AB, Weaver SC. Chikungunya virus-vector interactions. Viruses. 2014;6:4628-4663.

56. Diallo D, Sall AA, Buenemann M, Chen R, Faye O, Diagne CT, et al. Landscape Ecology of Sylvatic Chikungunya Virus and Mosquito Vectors in Southeastern Senegal. PLoS Negl Trop Dis. 2012;6:e1649.

57. Diallo M, Thonnon J, Traore-Lamizana M, Fontenille D. Vectors of Chikungunya virus in Senegal: current data and transmission cycles. Am J Trop Med. 1999; 60:281-286.

58. Felsenfeld AD. Chikungunya viruses in Thailand. Proc 7^th^ International Congress of Tropical Medicine and Malaria. p 1963;340.

59. Bennett KE, Olson KE, de Lourdes Muñoz M, Fernandez-Salas I, Farfan-Ale JA, Higgs S, et al. Variation in vector competence for dengue 2 virus among 24 collections of *Aedes aegypti* from Mexico and the United States. Am J Trop Med. 2002;67:85-92.

60. Boromisa RD, Rai KS, Grimstad PR. Variation in the vector competence of geographic strains of *Aedes albopictus* for dengue 1 virus. J Am Mosq Control Assoc. 1987;3:378-386.

61. Mavale MS, Ilkal MA, Dhanda V. Experimental studies on the susceptibility of Aedes vittatus to dengue viruses. Acta Virol. 1992;36:412-6.

62. Poole-Smith BK, Hemme RR, Delorey M, Felix G, Gonzalez AL, Amador M, et al. Comparison of vector competence of *Aedes mediovittatus* and *Aedes aegypti* for dengue virus: implications for dengue control in the Caribbean. PLoS Neg Trop Dis. 2015;9:e0003462.

63. Gubler DJ, Novak RJ, Vergne E, Colon NA, Velez M, Fowler J. *Aedes* (Gynometopa) *mediovittattus* (Diptera: Culicidae), a potential maintenance vector of dengue viruses in Puerto Rico. J Med Entomol. 1985;22:469-475.

64. Mitchell CJ, Miller BR, Gubler DJ. Vector competence of *Aedes albopictus* from Houston, Texas, for dengue serotypes 1 to 4, yellow fever, and Ross River viruses. J Am Mosq Control Assoc. 1987;3:460-465.

65. Medlock JM, Hansford KM, Schaffner F, Versteirt V, Hendrickx G, Zeller H, et al. A review of the invasive mosquitoes in Europe: ecology, public health risks, and control options. Vector Borne Zoonotic Dis. 2012;12:435-47.

66. Vezzani D, Eiras DE, Wisnivesky C. Dirofilariasis in Argentina: historical review and first report of *Dirofilaria immitis* in a natural mosquito population. Vet Parasitol. 2006;136:259-273.

67. Cancrini G, Scaramozzino P, Gabrielli S, Di Paolo M, Toma L, Romi R. *Aedes albopictus* and *Culex pipiens* implicated as natural vectors of *Dirofilaria repens* in central Italy. J Med Entomol. 2007;44:1064-1066.

68. Morchón R, Carretón E, González-Miguel J, Mellado-Hernández I. Heartworm diseases (*Dirofilaria immitis*) and their vectors in Europe – new distribution trends. Front Physiol. 2012;3:196.

69. Azari-Hamidian S, Yaghoobi-Ershadi MR, Javadian E, Abai MR, Mobedi I, Linton YM, et al. Distribution and ecology of mosquitoes in a focus of dirofilariasis in northwestern Iran, with the first finding of filarial larvae in naturally infected local mosquitoes. Med Vet Entomol. 2009;23:111-121.

70. Cancrini G, Scaramozzino P, Gabrielli S, Di Paolo M, Toma L, Romi R. *Aedes albopictus* and *Culex pipiens* implicated as natural vectors of *Dirofilaria repens* in central Italy. J Med Entomol. 2007;44:1064-1066.

71. Davis WA. A study of birds and mosquitoes as hosts for the virus of eastern equine encephalomyelitis. Am J Epidemiol.1940;32:45-59.

72. Armstrong PM, Andreadis TG. Eastern equine encephalitis virus in mosquitoes and their role as bridge vectors. Emerg Infect Dis. 2010;16:1869-1874.

73. Vasconcelos PF, Travassos da Rosa AP, Rodrigues SG, Travassos da Rosa ES, Dégallier N, Travassos da Rosa JF. Inadequate management of natural ecosystem in the Brazilian Amazon region results in the emergence and reemergence of arboviruses. Cadernos de Saúde Pública. 2001;17:155-164.

74. Hassan HK, Cupp EW, Hill GE, Katholi CR, Klingler K, Unnasch TR. Avian host preference by vectors of eastern equine encephalomyelitis virus. Am J Trop Med. 2003;69:641-647.

75. Walder R, Suarez OM, Calisher CH. Arbovirus studies in the Guajira region of Venezuela: activities of eastern equine encephalitis and Venezuelan equine encephalitis viruses during an interepizootic period. Am J Trop Med. 1984;33:699-707.

76. Mitchell CJ, Niebylski ML, Smith GC, Karabatsos N, Martin D, Mutebi JP, et al. Isolation of eastern equine encephalitis virus from *Aedes albopictus* in Florida. Science. 1992;257:526-7.

77. Vaidyanathan R, Edman JD, Cooper LA, Scott TW. Vector competence of mosquitoes (Diptera: Culicidae) from Massachusetts for a sympatric isolate of eastern equine encephalomyelitis virus. J Med Entomol. 1997;34:346-352.

78. Cupp EW, Klingler K, Hassan HK, Vlguers LM, Unnasch TR. Transmission of eastern equine encephalomyelitis virus in central Alabama. Am J Trop Med. 2003;68:495-500.

79. Srihongse S, Galindo P. The isolation of eastern equine encephalitis virus from *Culex* (Melanoconion) *taeniopus* Dyar and Knab in Panama. Mosq News. 1967;27:74-76.

80. Crans WJ. (2022, May) *Culiseta morsitans* (Theobald). (http://vectorbio.rutgers.edu/outreach/species/mors.htm)

81. Turell MJ, O'Guinn ML, Dohm D, Zyzak M, Watts D, Fernandez R, et al. Susceptibility of Peruvian mosquitoes to eastern equine encephalitis virus. J Med Entomol. 2008;45:720-725.

82. Cupp EW, Klingler K, Hassan HK, Viguers LM, Unnasch TR. Transmission of eastern equine encephalomyelitis virus in central Alabama. Am J Trop Med. 2003;68:495-500.

83. Turell MJ, O’Guinn ML, Jones JW, Sardelis MR, Dohm DJ, Watts DM, et al. Isolation of viruses from mosquitoes (Diptera: Culicidae) collected in the Amazon Basin region of Peru. J Med Entomol. 2005;42:891-898.

84. Coffey L, Crawford , Dee J, Miller R, Freier J, Weaver S. Serologic evidence of widespread Everglades virus activity in dogs, Florida. Emerg Infect Dis. 2006;12:1873-1879.

85. Dandawate CN, Rajagopalan PK, Pavri KM, Work TH. Virus isolations from mosquitoes collected in North Arcot district, Madras state, and Chittoor district, Andhra Pradesh between November 1955 and October 1957. Ind J Med Res. 1969;57:1420-1426.

86. Kokernot RH, Smithburn KC, Paterson HE, McIntosh BM. 1960. Isolation of Germiston virus, a hitherto unknown agent, from Culicine mosquitoes, and a report of infection in two laboratory workers. Am J Trop Med. 1960;9:62-69.

87. McIntosh BM, Jupp PG, Dos Santos IS, Meenehan GM. *Culex* (Eumelanomyia) *rubinotus* Theobald as vector of Banzi, Germiston and Witwatersrand viruses. I. Isolation of virus from wild populations of *C. rubinotus*. J Med Entomol. 1976;12:637-640.

88. Xue-Dong L, Fu-Xi Q, Huo Y, Yi-Nian R, Calisher CH. Isolation of Getah virus from mosquitoes collected on Hanian Island, China and results of a serosurvey. SE Asean J Trop Med. 1992;23:730-734.

89. Kay BH, Garley JG, Filippich C. The multiplication of Queensland and New Guinean arboviruses in *Aedes funereus* (Theobald) (Diptera: Guligidae). J Med Entomol. 1977;13:451-453.

90. Kay BH, Carley JG, Filippich C. The multiplication of Queensland and New Guinean arboviruses in *Culex annulirostris* Skuse and *Aedes vigilax* (Skuse) (Diptera: Culicidae). J Med Entomol. 1975;12:279-283.

91. Xia H, Wang Y, Atoni E, Zhang B, Zhimig Y. Mosquito associated viruses in China. Virol Sin. 2018;33:5-20.

92. Matsuyama T, Nakamura T, Isahai K, Oya A, Kobayashi M. Haruna virus, a group A arbovirus isolated from swine in Japan. Gumma J Med Sci. 1967;16:131-134.

93. Doherty RL, Gorman BM, Whitehead RH, Carley JG. Studies of arthropod-borne virus infections in Queensland. V. Survey of antibodies to group A arboviruses in man and other animals. Aust J Med Sci. 1966;44:365-378.

94. Ksiazek TG, Trosper JH, Cross JH, Basaca-Sevilla V. Isolation of Getah virus from Nueva Ecija Province, Republic of the Philippines. Trans R Soc Trop Med Hyg. 1981;75:312-3.

95. Shope RE. Bunyaviruses. In S. Baron (ed.). Medical Microbiology. 4^th^ edition. University of Texas Medical Branch at Galveston, Galveston, TX. 1996;56.

96. Toda A, Shope RE. Transmission of Guamá and Oriboca viruses by naturally infected mosquitoes. Nature. 1965;208:304.

97. Galindo P, Srihongse S. Transmission of arboviruses to hamsters by the bite of naturally infected *Culex* (Melanoconion) mosquitoes. Am J Trop Med. 1967;16:525-530.

98. Roca-Garcia M. The isolation of three neurotropic viruses from forest mosquitoes in eastern Colombia. J Infect Dis. 1944;75, 160–169.

99. Sirivanakarn S, Degallier N. Redescription of *Culex* (Melanoconion) *portesi* Senevet and Abonnenc, 1941, with notes on synonimy (Diptera: Culicidae). Mosq Syst. 1981;13:153-167.

100. Whitman, L. Personal communication. 1960. Collected from: Guaroa Virus. Arbovirus Catalog. Centers for Disease Control and Prevention.

101. Hayes CG, Corristan EC. A comparison of suckling mouse and mosquito succestibilty to infection by the Bunyamwera group viruses. Mosq News 1972;32:172-176.

102. Centers for Disease Control and Prevention (CDC). Guaroa Virus Arbovirus Catalog.

103. Lee VH, Sanmartin C. Isolations of Guaroa virus from Anopheles (Kerteszia) neivai in the Pacific lowlands of Colombia. Am J Trop Med. 1967;16:778-781.

104. Laemmert HW, Hughes TP. The virus of Ilhéus encephalitis; isolation, serological specificity and transmission. J Immunol. 1974;55:61-67.

105. Aitken THG, Anderson CR. Virus transmission studies with Trinidadian mosquitoes. Part II. Further observations. Am J Trop Med. 1959;8:41-45.

106. Lwande OW, Bucht G, Ahlm C, Ahlm K, Näslund J, Evander M. Mosquito-borne Inkoo virus in northern Sweden – isolation and whole genome sequencing. Virol J. 2017;14:61.

107. Brummer-Korvenkontio M, Saikku P, Korhonen P, Ulmanen I, Reunala T, Karvonen J. Arboviruses in Finland. IV. Isolation and characterization of Inkoo virus, a Finnish representative of the California group. Am J Trop Med. 1973;22:404-413.

108. Bulychev VP, Alekseev AN, Kostiukov MA, Gordeeva ZE, L'vov DK. Issyk-Kul virus transmission by *Aedes caspius caspius* Pall. mosquitoes via experimental bite. Med Parazitol. 1979;48:53-6.

109. Sallum MA, Forattini OP. Revision of the Spissipes section of *Culex* (Melanoconion) (Diptera: Culicidae). J Am Mosq Control Assoc. 1996;12:517-600.

110. Kramer LD, Bowen MD, Hardy JL, Reeves WC, Presser SB, Eldridge BF. Vector competence of alpine, Central Valley, and coastal mosquitoes (Diptera: Culicidae) from California for Jamestown Canyon virus. J Med Entomol. 1993;30:398-406.

111. Boromisa RD, Grimstad PR. Virus-vector-host relationships of Aedes stimulans and Jamestown Canyon virus in a northern Indiana enzootic focus. Am J Trop Med. 1986;35:1285-1295.

112. Sprance HE, Main AJ, Wallis RC, Elston J. Jamestown Canyon virus in Connecticut. Mosq News. 1979;38:392-395.

113. Campbell GL, Eldridge BF, Reeves WC, Hardy JL. Isolation of Jamestown Canyon virus from boreal Aedes mosquitoes from the Sierra Nevada of California. Am J Trop Med. 1991;44:244-249.

114. Boromisa RD, Grayson MA. Incrimination of *Aedes provocans* as a vector of Jamestown Canyon virus in an enzootic focus of northeastern New York. J Am Mosq Control Assoc. 1990;6:504-509.

115. Heard PB, Zhang MB, Grimstad PR. Laboratory transmission of Jamestown Canyon and snowshoe hare viruses (Bunyaviridae: California serogroup) by several species of mosquitoes. J Am Mosq Control Assoc. 1991;7:94-102.

116. Huang CH. Studies on factors as causes of inapparent infection in Japanese B encephalitis: virus strain, viremia, stability to heat and infective dosage. Acta Virol. 1957;1:36–45.

117. Ruben R, Tewari SC, Hiriyan J, Akiyama J. Illustrated keys to species of *Culex* (Culex) associated with Japanese encephalitis in Southeast Asia (Diptera: Culicidae). Mosq Syst. 1994;26:75-96.

118. Le Flohic G, Porphyre V, Barbazan P, Gonzalez JP. Review of climate landscape, and viral genetics as drivers of the Japanese encephalitis virus ecology. PLoS Neg Trop Dis. 2013;7:e2208.

119. Dhanda V, Kaul HN. Mosquito vectors of Japanese encephalitis virus and their bionomics in India. Proc Natl Sci India A. 1980;46: 759-768.

120. van den Hurk AF, Pyke AT, Mackenzie JS, Hall-Mendelin S, Ritchie SA. Japanese Encephalitis Virus in Australia: From Known Known to Known Unknown. Trop Med Infect Dis. 2019;4:38.

121. Vythilingam I, Oda K, Mahadevan S, Abdullah G, Thim CS, Hong CC, et al. Abundance, parity, and Japanese encephalitis virus infection of mosquitoes (Diptera: Culicidae) in Sepang District, Malaysia. J Med Entomol. 1997;34: 257-262.

122. Foote RH, Cook DR. Mosquitoes of Medical Importance. Washington DC (USA): United States Department of Agriculture. 1959.

123. Le Flohic G, Porphyre V, Barbazan P, Gonzalez JP. Review of climate landscape, and viral genetics as drivers of the Japanese encephalitis virus ecology. PLoS Neg Trop Dis. 2013;7:e2208.

124. Vythilingam I, Oda K, Mahadevan S, Abdullah G, Thim CS, Hong CC, et al. Abundance, parity, and Japanese encephalitis virus infection of mosquitoes (Diptera: Culicidae) in Sepang District, Malaysia. J Med Entomol. 1997;34: 257-262.

125. Foote RH, Cook DR. Mosquitoes of Medical Importance. Washington DC (USA): United States Department of Agriculture. 1959.

126. Doherty RL, Standfast HA, Domrow R, Wetters EJ, Whitehead RH, Carley JG. Studies of the epidemiology of arthropod-borne virus infections at Mitchell River Mission, Cape York Peninsula, north Queensland. IV. Arbovirus infections of mosquitoes and mammals, 1967-1969. 1971;65:504-513.

127. Centers for Disease Control and Prevention (CDC). 2016. La Crosse encephalitis: transmission.

128. Watts DM, Grimstad PR, DeFoliart GR, Yuill TM, Hanson RP. Laboratory transmission of LaCrosse encephalitis virus by several species of mosquitoes. J Med Entomol. 1973;10:583-586.

129. Westby KM, Fritzen C, Paulsen D, Poindexter S, Moncayo AC. La Crosse encephalitis virus infection field-collected *Aedes albopictus*, *Aedes japonicus*, and *Aedes triseriatus* in Tennessee. J Am Mosq Control Assoc. 2015;31:233-241.

130. Thompson WH, Anslow RO, Hanson RP, Defoliart GR. La Crosse virus isolations from mosquitoes in Wisconsin, 1964-68. Am J Trop Med. 1972;21:90-6.

131. Kokernot RH, McIntosh BM, Worth CB, Moraist DE, Weinbren MP. Isolation of viruses from mosquitoes collected at Lumbo, Mozambique. I. Lumbo virus, a new virus isolated from *Aedes* (Skusea) *pembaensis* Theobald. Am J Trop Med. 1962;11:678-682.

132. Kambris Z, Cook PE, Phuc HK, Sinkins SP. Immune activation by life-shortening *Wolbachia* and reduced filarial competence in mosquitoes. Science. 2009;326:134-136.

133. Onapa AW, Simonsen PE, Pederson EM, Okello DO. Lymphatic filariasis in Uganda: baseline investigations in Lira, Soroti and Katakwi districts. Trans R Soc Trop Med Hyg. 2001;95:161-167.

134. Rao RU, Atkinson LJ, Ramzy RM, Helmy H, Farid HA, Bockarie MJ, et al. A real-time PCR-based assay for detection of *Wuchereria bancrofti* DNA in blood and mosquitoes. Am J Trop Med. 2006;74: 826-832.

135. Hay SI, Sinka ME, Okara RM, Kabaria CW, Mbithi PM, Tago CC, et al. Developing global maps of the dominant *Anopheles* vectors of human malaria. PLoS Med. 2010;7:e1000209

136. Hanafi-Bojd AA, Vatandoost H, Jafari R. Susceptibility status of *Anopheles dthali* and An. *fluviatilis* to commonly used larvicides in an endemic focus of malaria, southern Iran. J Vect Borne Dis. 2006;43:34-38.

137. Rosen L, Reeves WC. Studies on avian malaria in vectors and hosts of encephalitis in Kern County, California. III. The comparative vector ability of some of the local culicine mosquitoes. Am J Trop Med. 1954;3:704-708.

138. Sinka ME, Bangs MJ, Manguin S, Coetzee M, Mbogo CM, Hemingway J, et al. The dominant *Anopheles* vectors of human malaria in Africa, Europe and the Middle East: occurrence data, distribution maps and bionomic précis. Parasit Vectors. 2010;3:117.

139. Deane LM. Malaria vectors in Brazil. Mem Inst Oswaldo Cruz. 1986;81:5-14.

140. Mendis C, Jacobsen JL, Gamage-Mendis A, Bule E, Dgedge M, Thompson R, et al. *Anopheles arabiensis* and *An. funestus* are equally important vectors of malaria in Matola coastal suburb of Maputo, southern Mozambique. Med Vet Entomol. 2000;14:171-180.

141. Antonio-nkondjio C, Kerah CH, Simard F, Awono-ambene P, Chouaibou M, Tchuinkam T, et al. Complexity of the malaria vectorial system in Cameroon: contribution of secondary vectors to malaria transmission. J Med Entomol. 2006;43:1215-1221.

142. Zaim M, Subbarao SK, Manouchehri AV, Cochrane AH. Role of *Anopheles culicifacies* s.l. and *An. pulcherrimus* in malaria transmission in Ghassreghand (Baluchistan), Iran. J Am Mosq Control Assoc. 1993;9: 23-26.

143. de Arruda M, Carvalho MB, Nussenzweig RS, Maracic M, Ferreira AW, Cochrane AH. Potential vectors of malaria and their different susceptibility to *Plasmodium falciparum* and *Plasmodium vivax* in northern Brazil identified by immunoassay. Am J Trop Med. 1986;35:873-881.

144. Reeves WC, Herold RC, Rosen L, Brookman B, Hammon WM. Studies on avian malaria in vectors and hosts of encephalitis in Kern County, California. II. Infections in mosquito vectors. Am J Trop Med. 1954;3:696-703.

145. Coggeshall LT. Infection of *Anopheles quadrimaculatus* with *Plasmodium cynomolgi*, a monkey malaria parasite, and with *Plasmodium lophurae*, an avian malaria parasite. Am J Trop Med. 1941;1:525-530.

146. Rickman LS, Jones TR, Long GW, Paparello S, Schneider I, Paul CF, et al. *Plasmodium falciparum*-infected *Anopheles stephensi* inconsistently transmit malaria to humans. Am J Trop Med. 1990;43:441-445.

147. Acosta-Ampudia Y, Monsalve DM, Rodríguez Y, Pacheco Y, Anaya JM, Ramírez-Santana C. Mayaro: An Emerging Viral Threat. Emerg Microbes Infect. 2018;7:1–11.

148. Long KC, Ziegler SA, Thangamani S, Hausser NL, Kochel TJ, Higgs S, et al. Experimental transmission of Mayaro virus by *Aedes aegypti*. Am J Trop Med. 2011;85: 750-757.

149. Smith GC, Francy DB. Laboratory studies of a Brazilian strain of *Aedes albopictus* as a potential vector of Mayaro and Oropouche viruses. J Am Mosq Control Assoc.1991;7:89-93.

150. Brustolin M, Pujhari S, Henderson CA, Rasgon JL. *Anopheles* mosquitoes may drive invasion and transmission of Mayaro virus across geographically diverse regions. PLoS Neg Trop Dis. 2018;12:e0006895.

151. Wiggins K, Eastmond B, Alto BW. Transmission potential of Mayaro virus in Florida *Aedes aegypti* and *Aedes albopictus* mosquitoes. Med Vet Entomol. 2018;32:436-442.

152. Muñoz M, Navarro JC. Mayaro virus: a re-amerging arbovirus in Venezuela and Latin America. Biomedica. 2012;32:286-302.

153. Dick GWA. Epidemiological notes on some viruses isolated in Uganda (Yellow fever, Rift Valley fever, Bwamba fever, West Nile, Mengo, Semliki forest, Bunyamwera, Ntaya, Uganda S and Zika viruses). Trans R Soc Trop Med Hyg. 1953;47:13-48.

154. Dick GW, Best AM, Haddow AJ, Smithburn KC. Mengo Encephalomyelitis. A hitherto unknown Virus affecting Man. Lancet. 1948;386-389.

155. Kokernot RH, Smithburn KC, de Meillon B, Paterson HE. Isolation of Bunyamwera virus from a naturally infected human being and further isolations from *Aedes* (Banksinelld) *circumluteolus* Theo. Am J Trop Med. 1958;7:579-84.

156. Worth C, Paterson HE, de Meillon B. The incidence of arthropod-borne viruses in a population of culicine mosquitoes in Tongaland, Union of South Africa (January 1956, through April 1960). Am J Trop Med. 1961;10:583-592.

157. Cornet M, Robin Y, Adam C, Valade M, Calvo MA. Transmission expérimentale comparée du virus amaril et du virus Zika chez *Aedes aegypi* (L). Cah ORSTOM Sér Ent Méd Parasitol. 1979;17:47–53.

158. McLean DM. Vectors of Murray Valley Encephalitis. J Infect Dis. 1957;223-227.

159. Woodall JP. Virus research in Amazonia. Atas Simp Biota Amazôn. 1967;6:31-63.

160. Mackenzie JS, Lindsay MD, Coelen RJ, Broom AK, Hall RA, Smith DW. Arboviruses causing human disease in the Australasian zoogeographic region. Arch Virol. 1994;136:447-67.

161. Dias, HG, dos Santos FB, Pauvolid-Corrêa A. An overview of neglected Orthobunyaviruses in Brazil. Viruses. 2022;14:987.

162. Demenev VA, SIa G, Roslaia IG, Obukhova VR, Koninskaia AI. Isolation of strains of Negishi virus in Khabarovsk Territory. Voprosy Virusol. 1987;32:105-8.

163. Belem Virus Laboratory, Brazil. Unpublished data. Collected from: Nepuyo Virus. Arbovirus Catalog. Centers for Disease Control and Prevention. 1965.

164. Whitman, L. Personal communication. Collected from: Nepuyo Virus. Arbovirus Catalog. Centers for Disease Control and Prevention.

165. Spence L, Anderson CR, Aitken THG, Downs WG. Nepuyo virus, a new group C agent isolated in Trinidad and Brazil. I. Isolation and properties of the Trinidadian strain. Am J Trop Med. 1966;15:71-4.

166. Centers for Disease Control and Prevention (CDC). Nepuyo Virus. Arbovirus Catalog.

167. Cupp EW, Scherer WF, Lok JB, Brenner RJ, Dziem GM, Ordonez JV. Entomological studies at an enzootic Venezuelan equine encephalitis virus focus in Guatemala, 1977-1980. Am J Trop Med. 1986;35:851-9.

168. Deardorff ER, Estrada-Franco JG, Freier JE, Navarro Lopez R, Travassos Da Rosa A, Tesh RB, et al. Candidate vectors and rodent hosts of Venezuelan equine encephalitis virus, Chiapas, 2006-2007. Am J Trop Med. 2011;85:1146-1153.

169. Monath TP. The Arboviruses:: Epidemiology and Ecology. CRC Press. 2020;1.

170. Williams MC, Woodall JP, Corbet PS. Nyando virus: a hitherto undescribed virus isolated from *Anopheles funestus giles* collected in Kenya. Arch Virol. 1965;15:422-427.

171. Ardoin PM, Simpson DI. Relations antigéniques entre le virus Nyando et deux viruse isolés en Ethiopie a partir de collectes d'Eretmapodites. Bull Soc Pathol Exot Filiales. 1965;58:573–589.

172. Turell MJ, Lundström JO. Effect of environmental temperature on the vector competence of *Aedes aegypti* and *Ae. taeniorhynchus* for Ockelbo virus. Am J Trop Med. 1990;43,543-550.

173. Lundström JO, Turell MJ, Niklasson B. Effect of environmental temperature on the vector competence of *Culex pipiens* and *Cx. torrentium* for Ockelbo virus. Am J Trop Med. 1990;43:534-542.

174. Turell MJ, LundstrÖM JO, Niklasson B. Transmission of Ockelbo Virus by *Aedes cinereus*, *Ae. communis*, and *Ae. excrucians* (Diptera: Culicidae) collected in an Enzootic Area in Central Sweden. J Med Entomol. 1990;27:266-268.

175. Francy DB, Jaenson TG, Lundström JO, Schildt EB, Espmark A, Henriksson B, et al. Ecologic studies of mosquitoes and birds as hosts of Ockelbo virus in Sweden and isolation of Inkoo and Batai viruses from mosquitoes. Am J Trop Med. 1989;41:355-363.

176. Gould E, Pettersson J, Higgs S, Charrel R, de Lamballerie X. Emerging arboviruses: Why today? One Health. 2017;4:1-13.

177. Haddow AJ, Davies CW, Walker AJ. O’nyong-nyong fever: an epidemic virus disease in East Africa 1. Introduction Trans R Soc Trop Med Hyg. 1960;54:517-522

178. Johnson BK, Gichogo A, Gitau G, Patel N, Ademba G, Kirui R, et al. Recovery of O’nyong nyong virus from *Anopheles funestus*, in western Kenya. Trans R Soc Med Hyg. 1981;75:239–241.

179. Smith GC, Francy DB. Laboratory studies of a Brazilian strain of *Aedes albopictus* as a potential vector of Mayaro and Oropouche viruses. J Am Mosq Control Assoc.1991;7:89-93.

180. Pinheiro F, Travassos da Rosa A, Travassos da Rosa J, Ishak R, Freitas RB, Gomes LMC, et al. Oropouche Virus I. A Review of Clinical, Epidemiological, and Ecological Findings. Am J Trop Med. 1981;30:149-160.

181. Aitken THG, Downs WG, Anderson CR, Spence L, Casals J. Mayaro Virus isolated from a Trinidadian Mosquito, Mansonia venezuelensis. Science 1960;131:986–986.

182. Tomori O, Aitken TH. Orungo virus: transmission studies with *Aedes albopictus* and *Aedes aegypti* (Diptera: Culicidae). J Med Entomol. 1978;14:523-6.

183. Cordellier R, Chippaux A, Monteny N, Heme G, Courtois B, Germain M et al. Isolements du virus Orungo à partir de femelles et de mâles d' *Aedes* selvatiques captures en Côte d’Ivoire. Entomol Med Parisitol. 1983;21:165-79.

184. Tomori O, Fabiyi A. Orungo virus: a new agent from mosquitoes and man in Uganda and Nigeria. Niger Med. 1977;7:5-8.

185. East Africa Virus Research Institute Annual Reports No 11-15, 1960-1965. Papers of Alexander John Haddow, 1912-1978, epidemiologist, Professor of Administrative Medicine, University of Glasgow, Scotland, 1971-1978. University of Glasgow Archive Services. GB 248 DC 068/4/3.

186. Renaudet, J, Robin, Y, Cornet, M, Coz, J. Recherches effectuées sur l'écologie des arbovirus au Sénégal. Rapport Annuel Centre Collaborateur OMS De Reference Et De Recherche Pour Les Arbovirus. Institut Pasteur De Dakar. 1976:3.

187. Germain, M, Herve, J-P, Geoffroy, B, Cornet, J-P. Department of Medical Entomology and Study of Virus Reservoirs.Annual Report, Institute Pastor of Bangui, 1975

188. Brown, SE, Morrison HG, Karabatsos N, Knudson, DL. Genetic relatedness of two new Orbivirus serogroups: Orungo and Lebombo. J Gen Virol. 1991;72:1065-1072.

189. McIntosch, BM, Jupp, PG, De Sousa J. Mosquitoes feeding at two horizontal levels in gallery forest in Natal, South Africa. with reference to possible vectors of chikungunya virus. Afr Entomol. 1972;35:81-90

190. Rapport Annuel. Centre Collaborateur OMS De Reference Et De Recherche Pour Les Arbovirus. Institut Pasteur, Dakar. 1984.

191. Kislenko GS, Chunikhin SP, Rasnitsyn SP, Kurenkov VB, Izotov VK. Reproduction of Powassan and West Nile viruses in *Aedes* *aegypti* mosquitoes and their cell culture. Med Parazitol. 1982;51:13-5

192. Jonkers AH, Metselaar D, de Andrade AH, Tikasingh ES. Restan virus, a new group C arbovirus from Trinidad and Surinam. Am J Trop Med. 1967;16:74–78.

193. Tantely LM, Boyer S, Fontenille D. A Review of Mosquitoes Associated with Rift Valley Fever Virus in Madagascar. Am J Trop Med. 2015;92:722-9.

194. Gear J, de Meillon B, Le Roux AF, Kofsky R, Rose-Innes R, Steyn JJ, et al. Rift Valley fever in South Africa. A study of the 1953 outbreak in the Orange Free State, with special reference to vectors and possible reservoir hosts. S Afr Med J. 1955;29:514–518.

195. Jupp P, Cornel A, Turell M, Bailey C, Beaman J. Vector competence tests with Rift Valley fever virus and five south African species of mosquito. Am Mosq Control Assoc. 1988;4:4–8.

196. Fontenille D. New Vectors of Rift Valley Fever in West Africa. Emerg Infect Dis. 1998;4:289–293.

197. Jupp PG, Kemp A, Grobbelaar A, Leman P, Burt FJ, Alahmed AM, et al. The 2000 epidemic of Rift Valley fever in Saudi Arabia: mosquito vector studies. Med Vet Entomol. 2002;16:245-52.

198. Turell MJ, Presley SM, Gad AM, Cope SE, Dohm DJ, Morrill JC, et al. Vector competence of Egyptian mosquitoes for Rift Valley fever virus. Am J Trop Med. 1996;54:136–139.

199. Jupp PG, Cornel AJ. Vector competence tests with Rift Valley fever virus and five south African species of mosquito. J Am Mosq Control Assoc. 1988;4:4-8.

200. Turell MJ, Linthicum KJ, Patrican LA, Davies FG, Kairo A, Bailey CL. Vector competence of selected African mosquito (Diptera: Culicidae) species for Rift Valley Fever Virus. J Med Entomol. 2008;45:102–108.

201. Mitchell CJ. Rocio encephalitis. In Service, MW Encyclopedia of Arthropod-transmitted Infections of Man and Domesticated Animals. CABI. 2001;434–7.

202. Mitchell CJ, Monath TP, Cropp CB. Experimental transmission of Rocio virus by mosquitoes. Am J Trop Med. 1981;30:465-72.

203. Mitchell CJ, Forattini OP, Miller BR. Vector competence experiments with Rocio virus and three mosquito species from the epidemic zone in Brazil. Rev Saúde Pública. 1986;20:171-177.

204. Claflin SB, Webb CE. Ross River Virus: Many Vectors and Unusual Hosts Make for an Unpredictable Pathogen. PLoS Path. 2015;11:e1005070.

205. Kay BH, Miles JAR, Gubler DJ, Mitchell CJ. Vectors of Ross River virus: An overview. In: JS Mackenzie, (ed.). Viral diseases in Southeast Asia and the Western Pacific. Academic Press. 1982;532-536.

206. Watson TM, Kay BH. Vector competence of *Aedes notoscriptus* (Diptera: Culicidae) for Ross River virus in Queensland, Australia. J Med Entomol. 1998;35:104-106.

207. Ballard JW, Marshall ID. An investigation of the potential of *Aedes camptorhynchus* (Thom.) as a vector of Ross River virus. Aust J Med Sci. 1986;64:197-200.

208. Jeffery JA, Ryan PA, Lyons SA, Kay BH. Vector competence of *Coquillettidia linealis* (Skuse) (Diptera: Culicidae) for Ross River and Barmah Forest viruses. Aust J Entomol. 2002;41:339-344.

209. Ryan PA, Do KA, Kay BH. Definition of Ross River virus vectors at Maroochy Shire, Australia. J Med Entomol. 2000;37:146-152.

210. Ritchie SA, Fanning ID, Phillips DA, Standfast HA, McGinn D, Kay BH. Ross River virus in mosquitoes (Diptera: Culicidae) during the 1994 epidemic around Brisbane, Australia. J Med Entomol. 1997;34:156-159.

211. Doherty RL, Carley JG, Filippich C, Kay BH, Gorman BM, Rajapaksa N. Isolation of sindbis (alphavirus) and leanyer viruses from mosquitoes collected in the northern territory of Australia, 1974. Aust J Exp Biol Med Sci. 1977;55:485–489

212. Hanafi HA, Fryauff DJ, Saad MD, Soliman AK, Mohareb EW, Medhat I, et al. Virus isolations and high population density implicate *Culex* *antennatus* (Becker) (Diptera: Culicidae) as a vector of Rift Valley Fever virus during an outbreak in the Nile Delta of Egypt. Acta Trop. 2011;119:119-124.

213. Davey MW, Mahon RJ, Gibbs AJ. Togavirus interference in *Culex annulirostris* mosquitoes. J Gen Virol. 1979;42:641-643.

214. Mathiot CC, Grimaud G, Garry P, Bouquety JC, Mada A, Daguisy AM, et al. An outbreak of human Semliki Forest virus infections in Central African Republic. Am J Trop Med. 1990;42:386-393.

215. Smithburn KC, Haddow AJ. Semliki forest virus. I. Isolation and pathogenic properties. J Immunol. 1944;49:141-57.

216. McIntosh BM, Worth CB, Kokernot RH. Isolation of semliki forest virus from *Aedes* (Aedimorphus) *argenteopunctatus* (theobald) collected in Portuguese East Africa. Trans R Soc Trop Med Hyg. 1961;55;192-198.

217. Gaĭdamovich S, Mel'nikova EE, Agafonov VI, Lokhova MD, Rodina V. Identification of a group A arbovirus isolated in the Far East. Vopr Virusol. 1975;3:317-320.

218. Macnamara FN. The susceptibility of chicks to Semliki Forest virus (Kumba strain). Ann Trop Med Parasitol. 1953;47:9-12.

219. Johansen CA, van den Hurk AF, Ritchie SA, Zborowski P, Nisbet DJ, Paru, Mackenzie, JS. Isolation of Japanese encephalitis virus from mosquitoes (Diptera: Culicidae) collected in the Western Province of Papua New Guinea, 1997-1998. 2000.

220. Shope RE. Epidemiology of other arthropod-borne flaviviruses infecting humans. Adv Virus Res. 2003;61:373-392

221. Rapport Annuel de I'Instiut Pasteur de Dakar.1980

222. Lwande OW, Näslund J, Lundmark E, Ahlm K, Ahlm C, Bucht G, et al. Experimental infection and transmission competence of Sindbis virus in *Culex torrentium* and *Culex pipiens* mosquitoes from northern Sweden. Vector-Borne and Zoonotic Dis. 2019;19:128-133.

223. Collins WE, Harrison AJ. Studies of Sindbis virus in *Anopheles* *albimanus* and *Aedes* *aegypti*. Mosq News. 1966;26.

224. Jupp PG, McIntosh PG. Quantitative experiments on the vector capability of *Culex* (Culex) *univittatus* Theobald with West Nile and Sindbis viruses. J Med Entomol. 1970;30: 371-373.

225. Jupp PG McIntosh BM. Quantitative experiments on the vector capability of *Culex* (Culex) *pipiens* *fatigans* Wiedemann with West Nile and Sindbis viruses. J Med Entomol. 1970;7:353-356.

226. Jupp PG, McIntosh PG, Blackburn NK. Experimental assessment of the vector competence of *Culex* (Culex) *neavei* Theobald with West Nile and Sindbis viruses in South Africa. Trans R Soc Trop Med Hyg. 1986;80:226-230.

227. Doherty RL, Carley JG, Mackerras MJ, Marks EN. Studies of arthropod-borne virus infections in Queensland. III. Isolation and characterization of virus strains from wild-caught mosquitoes in north Queensland. Aust J Med Sci. 1963;41:17-39.

228. JösT H, Bialonski A, Storch V, Günther S, Becker N, Schmidt-Chanasit J. Isolation and phylogenetic analysis of Sindbis viruses from mosquitoes in Germany. J Clin Mircobiol. 2010;48:1900–1903.

229. Taylor RM, Hurlbut HS, Work TH, Kingsbury JR, Frothingham TE. Sindbis virus: a newly recognized arthropod-transmitted virus. Am J Trop Med Hyg. 1955;4:844–6.

230. Rudnick A, Hammon WM, Sather GE. A strain of Sindbis virus isolated from *Culex bitaeniorhynchus* Mosquitoes in the Philippines, Am J Trop Med. 1962;11:546-549.

231. Bowen ETW, Simpson DIH, Platt GS, Way HJ, Smith CEG, Ching CY, et al. Arbovirus infections in Sarawak : the isolation of Kunjin virus from mosquitoes of the *Culex pseudovishnui* group. Ann Trop Med Parasitol. 1970;64:263-268.

232. East Africa Virus Research Institute Report. Government Printer, Nairobi, 1962;12.

233. McLean DM, Bergman SK, Gould AP, Grass P N, Miller MA, Spratt EE. California encephalitis virus prevalence throughout the Yukon Territory, 1971-1974. Am J Trop Med. 1975;24:676-684.

234. Hewlett MJ, Clerx JPM, Haaster CV, Chandler LJ, McLean DM, Beaty BJ. Genomic and biologic analyses of Snowshoe Hare virus field and laboratory strains. Am J Trop Med Hyg. 1992;46:524–532.

235. Iverson JO, Wagner RJ, DeJong C, McLintock J. California encephalitis virus in Sastchewan: isolation from boreal Aedes mosquitoes. Can J Public Health. 1973;64:590-59.

236. Newhouse VF, Burgdorfer W, Corwin D. Field and laboratory studies on hosts and vectors of Snowshoe Hare strain of California Virus. Mosq. News. 1971;31:401–408.

237. Bearcroft WG. Zika virus infection experimentally induced in a human volunteer. Trans R Soc Trop Med Hyg. 1956;50:442–8.

238. Haddow AD, Nasar F, Guzman H, Ponlawat A, Jarman RG, Tesh RB, et al. Genetic characterization of Spondweni and Zika viruses and susceptibility of geographically distinct strains of *Aedes aegypti*, *Aedes albopictus* and *Culex quinquefasciatus* (Diptera: Culicidae) to Spondweni virus. PLoS Neg Trop Dis. 2016;10:e0005083.

239. Macnamara FN. Zika virus: a report on three cases of human infection during an epidemic of jaundice in Nigeria. Trans R Soc Trop Med. 1954;48:39-145.

240. Brottes H, Rickenbach A, Bres P, Salaun J-J, Ferara L. Les arbovirus au Cameroun : isolements à partir de moustiques. Bull Org mond Santé. 1966;35:811-825

241. Centers for Disease Control and Prevention (CDC). 2009. St. Louis encephalitis: transmission.

242. Mitamura T, Kitaoka M, Watanabe S, Iwasaki T, Ishikawa I, Tenjin S, et al. Uber die Bedeutung der Mucken fur die Ubertragung verscheidener Enzephalitis-Virusarten. Trans Jap Pathol Soc. 1940;30:561-570

243. Sardelis MR, Turell MJ, Andre RG. Experimental transmission of St. Louis encephalitis virus by *Ochlerotatus j. japonicus*. J Am Mosq Control Assoc. 2003;19:159-162.

244. Hammon WM, Reeves WC. Laboratory transmission of St. Louis encephalitis virus by three genera of mosquitoes. J Exp Med. 1943;78:241-253.

245. Reisen WK, Fang Y, Martinez VM. Avian host and mosquito (Diptera: Culicidae) vector competence determine the efficiency of West Nile and St. Louis encephalitis virus transmission. J Med Entomol. 2005;42:367-375.

246. Chamberlain RW, Sudia WD, Gillett JD. St. Louis encephalitis virus in mosquitoes. Am J Hyg.1959;70:221-236.

247. Hardy JL, Rosen L, Kramer LD, Presser SB, Shroyer DA, Turell MJ. Effect of rearing temperature on transovarial transmission of St. Louis encephalitis virus in mosquitoes. Am J Trop Med. 1980;29: 963-968.

248. Turell MJ, O’Guinn ML, Dohm DJ, Jones JW. Vector competence of North American mosquitoes (Diptera: Culicidae) for West Nile virus. J Med Entomol. 2001;38:130-134.

249. Hardy JL, Rosen L, Reeves WC, Scrivani RP, Presser SB. Experimental transovarial transmission of St. Louis encephalitis virus by *Culex* and *Aedes* mosquitoes. Am J Trop Med. 1984;33:166-175.

250. Taylor DJ, Meadows KE, Lewis AL, Bond JO. Arbovirus vector surveillance following the 1962 St. Louis encephalitis epidemic in the Tampa Bay area. Mosq News. 1968;28:42-45.

251. Chamberlain RW, Sudia WD, Coleman PH, Beadle LD. Vector studies in the St. Louis encephalitis epidemic, Tampa Bay area, Florida, 1962. Am J Trop Med. 1964;13:457-461.

252. Sudia WD, Coleman PH, Chamberlain RW, Wiseman JS, Work TH. St. Louis encephalitis vector studies in Houston, Texas, 1964. J Med Entomol. 1967;4:32-36.

253. Hammon WM, Reeves WC, Brookman B, Izumi EM, Gjullin CM. Isolation of the viruses of western equine and St. Louis encephalitis from *Culex tarsalis* mosquitoes. Science. 1941;94:328-330.

254. Beranek MD, Gallardo R, Almiron WR, Contigiani MS. First detection of *Mansonia titillans* (Diptera: Culicidae) infected with St. Louis encephalitis virus (Flaviviridae: Flavivirus) and Bunyamwera serogroup (Peribunyaviridae: Orthobunyavirus) in Argentina. J Vect Ecol. 2018;43:340-3.

255. Lebl K, Silbermayr K, Obwaller A, Berer D, Brugger K, Walter M et al. Mosquitoes (Diptera: Culicidae) and their relevance as disease vectors in the city of Vienna, Austria. Parasitol Res. 2015;114:707-713.

256. Marhoul Z. Susceptibility of *Anopheles* *gambiae* mosquito cell line (MOS 55) to some arboviruses. Acta Virol. 1973;17:507-9.

257. Bulychev VP, Kostyukov MA, Gordeeva ZE. Experimental infection of *Aedes caspius* Pall mosquitoes with Tahyna virus. Med Parazitol. 1978;47:63-65.

258. Traavik T, Mehl R, Wiger R. California encephalitis group viruses isolated from mosquitoes collected in Southern and Arctic Norway. Acta Pathol Microbiol Scand B Microbiol. 1978;86:335–42.

259. Simková A, Danielová V, Bárdos V. Experimental transmission of the Tahyna virus by *Aedes vexans* mosquitoes. Acta Virol. 1960;4:341-347.

260. Danielová V, Málková D, Minár J, Ryba J. Dynamics of the natural focus of Tahyna virus in southern Moravia and species succession of its vectors, the mosquitoes of the genus *Aedes*. Folia Parasitol. 1976;23:243-249.

261. Bouloy M. 3-segment RNA genone of Lumbo-virus (bunyavirus) Intervirol. 1973;2:173.

262. L'vov DK, Shcherbin LD, Zairov GK, Artiukhov NI, L'vov SD. Isolation of a Tahyna-like virus (Bunyaviridae, Bunyavirus, California encephalitis complex) on northern Sakhalin Island. Voprosy Virusol. 1987;32:588-90.

263. Danielová V, Holubová J. Two more mosquito species proved as vectors of Tahyna virus in Czechoslovakia. Folia Parasitol. 1977;24:187-189.

264. Bárdoš V, Danielova V. The Tahyña virus-a virus isolated from mosquitoes in Czechoslovakia. J Hyg Epidemiol Microbiol Immunol. 1959;3.

265. Malkova D, Marhoul Z. Influence of temperature corresponding to that of the vector on Tahyna virus. Acta Virol. 1976;20:494-8.

266. Sudia WD, Coleman PH, Chamberlain RW. Experimental vector-host studies with Tensaw virus, a newly recognized member of the Bunyamwera arbovirus group. Am J Trop Med.. 1969;18(1):98-102.

267. Chamberlain RW, Sudia WD, Coleman PH. Isolations of an arbovirus of the Bunyamwera group (Tensaw virus) from mosquitoes in the southeastern United States, 1960-1963. Am J Trop Med. 1969;18.

268. Panday RS, Digoutte JP. Tonate and Guama-group viruses isolated from mosquitoes in both a savannah and coastal area in Surinam. Trop Geogr Med. 1979;31:275-82.

269. Andrews WN, Rowley WA, Wong YW, Dorsey DC, Hausler WJ. Isolation of Trivittatus Virus from larvae and adults reared from field-collected larvae of *Aedes trivittatus* (Diptera: Gulicidae). J Med Entomol. 1977;13:699-701.

270. Lewis AL, Hammon WM, Sather GE, Taylor DJ, Bond JO. Isolations of the California group arbovirus from Florida mosquitoes. Am J Trop Med. 1965;14.

271. Anderson JF, Main AJ, Armstrong PM, Andreadis TG, Ferrandino FJ. Arboviruses in North Dakota, 2003–2006. Am J Trop Med. 2015;92:377-393.

272. Bäckman S, Näslund J, Forsman M, Thelaus J. Transmission of tularemia from a water source by transstadial maintenance in a mosquito vector. Sci Rep. 2015;5:7793.

273. Lundström JO, Andersson A, Bäckman S, Schäfer ML, Forsman M, Thelaus J. Transstadial transmission of *Francisella tularensis holarctica* in mosquitoes, Sweden. Emerg Infect Dis. 2011;17:795-799.

274. Olsufiev NG. Parasitology of tularemia, In LM Khatenever (ed.). Tuleremia infection. Moscow, Russia. 1943;74-92.

275. Schaffner F, Angel G, Geoffroy B, Hervy J, Rhaiem A, Brunhes J. The mosquitoes of Europe. An identification and training programme. IRD Editions & EID Méditerranée. 2001.

276. Nikolay B, Diallo M, Faye O, Boye CS, Sall AA. Vector competence of *Culex neavei* (Diptera: Culicidae) for Usutu virus. Am J Trop Med. 2012;86:993.

277. Calzolari M, Bonilauri P, Bellini R, Albieri A, Defilippo F, Tamba M,et al. Usutu virus persistence and West Nile virus inactivity in the Emilia-Romagna region (Italy) in 2011. PLoS One. 2013;8:e63978.

278. Ndiaye EH, Diallo D, Fall G, Ba Y, Faye O, Dia I, et al. Arboviruses isolated from the Barkedji mosquito-based surveillance system, 2012-2013. BMC Infect Dis. 2018;18:1-4.

279. Woodall JP. The viruses isolated from arthropods at the East African Virus Research Institute in the 26 years ending December 1963. Proc E Afr Acad. 1964;2:141-6.

280. Vázquez A, Ruiz S, Herrero L, Moreno J, Molero F, Magallanes A, et al. West Nile and Usutu viruses in mosquitoes in Spain, 2008–2009. Am J Trop Med. 2011;85:178.

281. Mancini G, Garofalo G, Palmieri D, Santilli A, de Ascentis M, Quaglia M, et al. Usutu virus detection in Abruzzo region, Italy: the entomological surveillance as key tool for the mosquito-borne disease prevention. Int J Infect Dis. 2019;79:140.

282. Mannasse B, Mendelson E, Orshan L, Mor O, Shalom U, Yeger T, et al. Usutu virus RNA in mosquitoes, Israel, 2014–2015. Emerg Infect Dis. 2017;231699.

283. Jöst H, Bialonski A, Maus D, Sambri V, Eiden M, Groschup MH, et al. Isolation of usutu virus in Germany. Am J Trop Med. 2011;85:551–3

284. Weaver SC, Scherer WF, Cupp EW, Castello DA. Barriers to dissemination of Venezuelan encephalitis viruses in the Middle American enzootic vector mosquito, *Culex* (Melanoconion) *taeniopus*. Am J Trop Med. 1984;33:953-60.

285. Smith DR, Carrara AS, Aguilar PV, Weaver SC. Evaluation of methods to assess transmission potential of Venezuelan equine encephalitis virus by mosquitoes and estimation of mosquito saliva titers. Am J Trop Med. 2005;73:33-9.

286. Turell MJ, O’Guinn M, Olive Jr. Potential for New York mosquitoes to transmit West Nile virus. Am J Trop Med. 2000;62:413-414.

287. Smith DR, Adams AP, Kenney JL, Wang E, Weaver SC. Venezuelan equine encephalitis virus in the mosquito vector *Aedes* *taeniorhynchus*: infection initiated by a small number of susceptible epithelial cells and a population bottleneck. Virol. 2008;372:176-86.

288. Weaver SC, Salas R, Rico-Hesse R, Ludwig GV, Oberste MS, Boshell J, et al. Re-emergence of epidemic Venezuelan equine encephalomyelitis in South America. Lancet. 1996;348:436-440.

289. Thenmozhi V, Mariappan T, Krishnamoorthy R, Baskarn G, Krishnamoorthi R, Balaji T, et al. A first note on Japanese encephalitis virus isolation from *Culex quinquefasciatus* Say in Northern West Bengal. Int J Mosq Res. 2014;1:1-4.

290. Hayes CG. West Nile fever in: The arboviruses: epidemiology and ecology. TP Monath ed. CRC Press, Boca Raton, FL. 1989;5:59-88.

291. Dunphy BM, Kovach KB, Gehrke EJ, Field EN, Rowley WA, Bartholomay LC, et al. Long-term surveillance defines spatial and temporal patterns implicating *Culex tarsalis* as the primary vector of West Nile virus. Sci Rep. 2019;9:6637.

292. Hubálek Z, Halouzka J. West Nile fever—a reemerging mosquito-borne viral disease in Europe. Emerg. Infect. Dis. 1999;5:643-50.

293. Akhter R, Hayes CJ, Bagar S, Reisen WK. West Nile virus in Pakistan. III. Comparative vector capability of *Culex tritaeniorhynchus* and eight other species of mosquitoes. Trans R Soc Trop Med Hyg. 1982;76:449-53.

294. Philip CB, Smadel JE. Transmission of West Nile virus by infected *Aedes albopictus*. Proc R Soc B. 1943;53:49-50.

295. Goddard LB, Roth AE, Reisen WK, Scott TW. Vector competence of California mosquitoes for West Nile virus. Emerg Infect Dis. 2002;8:1385-1391.

296. Sardelis MR, Turell MJ. *Ochlerotatus j. japonicus* in Frederick County, Maryland: discovery, distribution, and vector competence for West Nile virus. J Am Mosq Control Assoc. 2001;17:127Ð141.

297. Kitaoka, M. Experimental transmission of the West Nile virus by the mosquito. Japan Med Assoc J. 1950;3:77-81.

298. Centers for Disease Control and Prevention (CDC). West Nile virus activity—eastern United States, 2001. MMWR. 2001;50: 617-619.

299. Centers for Disease Control and Prevention (CDC). Update: West Nile virus activity—northeastern United States, 2000. MMWR. 2000;49:820-822.

300. Anderson JF, Andreadis TG, Vossbrinck CR, Tirrell S, Wakem EM, French RA, et al. Isolation of West Nile virus from mosquitoes, crows, and a Cooper’s Hawk in Connecticut. Science. 1999;286:2331-3.

301. Nir Y, Goldwasser R, Lasowski Y, Margalit J. Isolation of West Nile virus strains from mosquitoes in Israel. Am J Epidemiol. 1968;87:496-501.

302. Granwehr BP, Lillibridge KM, Higgs S, Mason PW, Aronson JM, Campbell GA, et al. West Nile virus: where are we now? Lancet Infect Dis. 2004;4:547-56.

303. Hardy JL. The ecology of western equine encephalomyelitis virus in the Central Valley of California, 1945-1985. Am J Trop Med. 1987;37:18S-32S.

304. Minnesota Department of Health. Western equine encephalitis fact sheet. 2018.

305. Wang Z, Zhang X, Li C, Zhang Y, Xing D, Wu Y, et al. Vector competence of five common mosquito species in the People’s Republic of China for western equine encephalitis virus. Vector-Borne and Zoonotic Dis. 2012;12:605-608.

306. Miles JA, Pillai JS, Maguire T. Multiplication of Whataroa virus in mosquitoes. J Med Entomol. 1973;10:176-85.

307. Austin FJ. The arbovirus vector potential of a simuliid. Ann Trop Med Parasitol. 1967;61(2):189-99.

308. McIntosh BM, Kokernot RH, Paterson HE. Witwatersrand virus: an apparently new virus isolated from Culicine mosquitoes. S Afr Med J. 1960;25:33-7.

309. Groot H. Estudios sobre virus transmitidos por artrópodos en Colombia. Rev Acad Colomb Cien Exac Fis Nat. 1964;12:3-23.

310. Aitken TH, Spence L, Jonkers AH, Anderson CR. Wyeomyia-virus isolations in Trinidad, West Indies. Am J Trop Med.1968;17.

311. Tikchonenko TI. Comprehensive Virology: Newly Characterized Vertebrate Viruses. Heinz Fraenkel-Conrat H, Wagner RR (eds.). New York (NY): Plenum Press. 1975.

312. Whitman L, Antunes PCA. The transmission of two strains of jungle yellow fever virus by *Aedes aegypti*. Am J Trop Med. 1938;18:135-147.

313. Davis NC, Shannon RC. Studies on yellow fever in South America. Attempts to transmit the virus with certain Aedine and Sabethine mosquitoes and with *Triatomas* (Hemiptera). Am J Trop Med. 1931;11:21-29.

314. Whitman L, Antunes PCA. Studies on the capacity of various Brazilian mosquitoes representing the genera *Psorophora*, *Aedes*, *Mansonia*, and *Culex*, to transmit yellow fever. Am J Trop Med. 1937;17:803-823.

315. Davis NC, Shannon RC. Studies on yellow fever in South America: V. transmission experiments with certain species of *Culex* and *Aedes*. Exp Med. 1929;50:793-801.

316. Cornelius BP. Studies on transmission of experimental yellow fever by mosquitoes other than *Aedes*. Am J Trop Med. 1930;10:1-16.

317. Whitman L, Antunes PCA. The transmission of two strains of jungle yellow fever virus by *Aedes aegypti*. Am J Trop Med. 1938;18:135-147.

318. Beaty BJ, Aitken THG. In vitro transmission of yellow fever virus by geographic strains of *Aedes aegypti*. Mosq News. 1979;39:232-238.

319. Davis NC, Shannon RC. Studies on Yellow Fever in South America V. Transmission experiments with certain species of *Culex* and *Aedes*. Exp. Med. 1929;50:793-801.

320. Hartberg WK, Gerberg EJ. Laboratory colonization of *Aedes simpsoni* (Theobald) and *Eretmapodites quinquevittatus* Theobald. Bull World Health Organ. 1971;45:850.

321. Dinger JE, Schueiner WAP, Snijders EP, Swellengrebel NH. Onderzook over gele koorts in Nederland (derde medeeling). Ned Tijdschr Geneeskd. 1929;73:5982-91.

322. Bauer J. The transmission of yellow fever by mosquitoes other than *Aedes aegypti*. Am J Trop Med 1928;8:261-282.

323. Strode GK. Yellow Fever New York: McGraw-Hill Book Co; 1951.

324. C Cardoso JD, De Almeida MA, Dos Santos E, Da Fonseca DF, Sallum MA, Noll CA, et al. Yellow fever virus in *Haemagogus leucocelaenus* and *Aedes serratus* mosquitoes, southern Brazil, 2008. Emerg Infect Dis. 2010;16:1918.

325. de Rodaniche E, Galindo P, Johnson CM. Isolation of yellow fever virus from *Haemagogus lucifer*, *H. equinus*, *H. spegazzinii falco*, *Sabethes chloropterus* and *Anopheles neivai* captured in Panama in the fall of 1956. Am J Trop Med. 1957;6(4):681-5.

326. Aliota MT, Peinado SA, Osorio JE, Bartholomay LC. *Culex pipiens* and *Aedes triseriatus* mosquito susceptibility to Zika virus. Emerg Infect Dis. 2016;22:1857-1859.

327. Althouse BM, Vasilakis N, Sall AA, Diallo M, Weaver SC, Hanley KA. Potential for Zika virus to establish a sylvatic transmission cycle in the Americas. PLoS Neg Trop Dis. 2016;10:e0005055.

328. Benelli G, Romano D. Mosquito vectors of Zika virus. Entomol. Gen. 2017;36:309-318.

329. Diallo D, Sall AA, Diagne CT, Faye O, Faye O, Ba Y, et al. Zika virus emergence in mosquitoes in southeastern Senegal, 2011. PLoS One. 2014;9:e109442.

330. Richard V, Paoaafaite T, Cao-Lormeau VM. Vector competence of French Polynesian *Aedes aegypti* and *Aedes polynesiensis* for Zika virus. PLoS Neg Trop Dis. 2016;10:e0005024.

331. Ayres CF, Guedes DR, Paiva MH, Morais-Sobral MC, Krokovsky L, Machado LC, et al. Zika virus detection, isolation and genome sequencing through Culicidae sampling during the epidemic in Vitória, Espírito Santo, Brazil. Parasit Vectors. 2019;12:1-9.

332. Musso D, Gubler DJ. Zika virus. Clin Microbiol Rev. 2016;29:487-524.
