# Supplementary material for: Robust network stability of mosquitoes and human pathogens of medical importance
Source: Parasit Vectors. 2022 Jun 20;15:216. doi: 10.1186/s13071-022-05333-4 (PMC9208160; doi:10.1186/s13071-022-05333-4)
Supplement: Supplementary file 2 — Additional file 2: Table S2. Associations of vectors and pathogens from cluster analysis (Figs. 1, 2). Groups identified from analysis of known vectors only are identified in bold, with superscripts corresponding to cluster number. [file 13071_2022_5333_MOESM2_ESM.docx]

| **Cluster** | **Vectors** | **Pathogen(s)** |
| --- | --- | --- |
| 1 | *Taeniorhynchus africanus*, *Taeniorhynchus fuscopennatus*, *Taeniorhynchus uniformis* | Mengovirus |
| 2 | *Aedes argenteopunctatus*, *Aedes atlanticus*, *Aedes caballus*, *Aedes canator*, ***Aedes circumluteolus*^11^**, *Aedes cumminsii*, *Aedes dendrophilus*, *Aedes dentatus*, *Aedes excrucians*, *Aedes fitchii*, *Aedes fryeri*, *Aedes implicatus*, *Aedes infirmatus*, *Aedes juppi*, *Aedes lineatopennis*, ***Aedes mcintoshi*^14b^** , ***Aedes ochraceus*^14b^**, *Aedes palpalis*, *Aedes tarsalis*, *Aedes unidentatus*,  *Anopheles brohieri*, *Anopheles cincereus*, *Anopheles coustani*, *Anopheles crucians*, *Anopheles furcifer*, *Anopheles squamosus*,  *Coquillettidia fuscopennata*,  *Culex erraticus*, *Culex perexigus*, *Culex poicilipes*, *Culex territam*, *Culex zombaensis*,  *Culiseta minnesotae*,  *Eretmapodites quinquevittatus*, *Eretmapodites silvestris*,  ***Mansonia africana*^11^**, *Mansonia dyari*, ***Mansonia uniformis*^11^** | Bwamba, Middelburg, Pongola, **Rift Valley^14b^**, Shokwe, **Spondweni^11^** |
| 3 | *Aedes argyrothorax*, *Aedes scapularis*, *Aedes serratus*, *Aedes sexlineatus*,  *Aediomyia squamipinnis*,  *Anopheles nimbus*,  *Coquillettidia arribalzagai*, *Coquillettidia chrysonotum*,  *Culex amazonesis*, *Culex opisthopus*,  *Haemagogus leucocephalus*,  *Limatus asulleptus*, *Limatus flavisetosus*,  *Mansonia venezuelensis*,  *Ochlerotatus fulvus*,  *Psorophora albigenu*, *Psorophora albipes*, *Psorophora ferox*,  *Sabethes chloropterus*,  *Trichoprosopon digitatum*, *Trichoprosopon leucopus*, *Trichoprosopon longipes*,  *Wyeomyia aporonoma*, *Wyeomyia complosa* | Ilheus, Oropouche, Rocio, Wyeomyia virus |
| 4 | *Aedes abnormalis*, ***Aedes aegypti*^14a^**^,^**^15a^**^,^**^b^**^,^**^d^**^,^**^e^**, *Aedes africanus*, ***Aedes albopictus*^15a^**^,^**^b^**^,^**^c^**, *Aedes alternans*, *Aedes apicoannulatus*, *Aedes apicoargenteus*, *Aedes arborealis*, *Aedes calceatus*, ***Aedes camptorhynchus*^10b^**, *Aedes fluviatilis*, *Aedes fulgens*, *Aedes funereus*, *Aedes furcifer*, *Aedes hensilli*, *Aedes hirsutus*, *Aedes metallicus*, *Aedes multiplex*, ***Aedes notoscriptus*^10b^**, *Aedes opok*, *Aedes procax*, *Aedes scutellaris*, *Aedes septemstriatus*, *Aedes simpsoni*, *Aedes taylori*, *Aedes togoi*, *Aedes unilineatus*, ***Aedes vigilax*^10b^**, *Aedes vittatus*,  *Anopheles amictus*,  *Armigeres obturbans*, *Armigeres subalbatus*,  *Coquillettidia linealis*,  ***Culex annulirostris*^10a^**^,^**^b^**, *Culex australicus*, *Culex dolosus*, *Culex perfuscus*, *Culex sitiens*,  *Eretmapodites chrysogaster*,  *Ficalbia flavens*,  *Haemagogus capricorni*, *Haemagogus equinus*, ***Haemagogus janthinomys^8^***, *Haemagogus lucifer*, *Haemagogus mesodentatus*, *Haemagogus spegazzinii*,  *Mansonia septempunctata*,  *Ochlerotatus albofasciatus*, *Ochlerotatus crinifer*,  *Psorophora cyanescens* | Apeu, Barmah Forest, Caraparu Virus,  **Chikungunya^15a^**, **Dengue^15b^**, **Dirofilariasis^15c^**, Ganjam, Getah, Kokobera, **Mayaro^8^**, **Murray Valley Encephalitis^10a^**, Orungo, Powassan, Restan, **Ross River^10b^**, Semliki Forest, Sepik, **Yellow Fever (jungle)^15d^**, **Yellow Fever (urban)^15e^**, Zika |
| 5 | *Aedes dalzieli*, ***Aedes polynesiensis*^6^**, *Aedes tarsalis*,  ***Anopheles funestus*^9^** ^,^ ***Anopheles gambiae*^9^**,  *Mansonia aurites* | Ilesha, **Lymphatic Filariasis (elephantitis)^6^**, Nyando, **O'nyong'nyong^9^**, Tataguine |
| 6 | ***Anopheles aconitus*^7^**, ***Anopheles albimanus*^7^**, ***Anopheles albitarsis*^7^**, ***Anopheles annularis*^7^**, ***Anopheles aquasalis*^7^**, ***Anopheles arabiensis*^7^**, ***Anopheles argyritarsis*^7^**, ***Anopheles atroparvus*^7^**, ***Anopheles balabacensis*^7^**, ***Anopheles barbirostris*^7^**, ***Anopheles bellator*^7^**, ***Anopheles campestris*^7^**, *Anopheles carnevalei*, ***Anopheles cruzii*^7^**, ***Anopheles culicifacies*^7^**, ***Anopheles darlinging*^7^**, ***Anopheles dirus*^7^**, *Anopheles dthali*, ***Anopheles farauti*^7^**, ***Anopheles flavirostris*^7^**, ***Anopheles fluviatilis*^7^**, *Anopheles franciscanus*, ***Anopheles freeborni*^7^**, *Anopheles hancocki*, *A****nopheles koliensis*^7^**, ***Anopheles labranchiae*^7^**, ***Anopheles lesteri*^7^**, ***Anopheles letifer*^7^**, ***Anopheles leucosphyrus*^7^**, ***Anopheles maculatus*^7^**, ***Anopheles marajoara*^7^**, *Anopheles marshallii*, ***Anopheles melas*^7^**, *Anopheles merus*, *A****nopheles messeae*^7^**, ***Anopheles minimus*^7^**, ***Anopheles moucheti*^7^**, ***Anopheles multicolor*^7^**, ***Anopheles nigerrimus*^7^**, ***Anopheles nili*^7^**, ***Anopheles nuneztovari*^7^**, *Anopheles oswaldoi*, *Anopheles ovengensis*, *Anopheles paludis*, ***Anopheles pharoensis*^7^**, ***Anopheles pseudopunctipennis*^7^**, ***Anopheles pulcherrimus*^7^**, ***Anopheles punctimacula*^7^**, ***Anopheles punctulatus*^7^**, ***Anopheles quadrimaculatus*^7^**, ***Anopheles sacharovi*^7^**, ***Anopheles sergentii*^7^**, ***Anopheles sinensis*^7^**, ***Anopheles stephensi*^7^**, ***Anopheles subpictus*^7^**, ***Anopheles sundaicus*^7^**, ***Anopheles superpictus*^7^**, *Anopheles triannulatus*, *Anopheles wellcomei*, *Anopheles ziemanni* | **Malaria^7^** |
| 7 | *Aedes abserratus*, *Aedes cantans*, *Aedes caspius*, *Aedes cataphylla*, *Aedes cinereus*, ***Aedes communis*^4^**, *Aedes detritus*, *Aedes dianteus*, *Aedes hexodontus*, *Aedes intrudens*, *Aedes pembaensis*, *Aedes provocans*, *Aedes punctor*, *Aedes squamiger*, *Aedes sticticus*, *Aedes stimulans*, ***Aedes vexans*^14a^**^,^**^b^**,  *Anopheles claviger*, *Anopheles hrycanus*,  *Coquillettidia richiardii*,  *Culiseta annulata*, *Culiseta impatiens*, *Culiseta inornata*,  *Ochlerotatus excrucians* | **Inkoo^4^**, Issyk-Kul, Jamestown Canyon, Lumbo Virus, Negishi, Snowshoe hare, **Tahyna^12c^**, Tularemia |
| 8 | *Anopheles brasiliensis*, *Anopheles mediopunctatus*,  *Coquillettidia albicosta*, *Coquillettidia venezuelensis*,  *Culex aikenii*, *Culex crybda*, *Culex ocossa*, ***Culex portesi*^3^**, *Culex spissipes*,  *Culex vomerifer*, *Culex zeteki*,  *Deinocerites pseudes*,  *Limatus* sp.,  *Mansonia pseudotitillans*, *Mansonia* sp*.*, *Mansonia titillans*,  *Psorophora* sp.,  *Trichoprosopon* sp*.*,  *Wyeomyia melanocephala*, *Wyeomyia occulta*, *Wyeomyia pseudopecten* | Bussuquara, Catu virus, **Guama Virus^3^**, Madrid Virus, Marituba, Murutucu virus, Oriboca, Ossa, Tonate |
| 9 | *Aedes luteocephalus*, *Aedes mormanensis*, ***Aedes quasiunivittatus*^1^**,  *Anopheles maculipennis*,  *Coquillettidia aurites*,  *Culex hortensis*, *Culex nakuruensis*, *Culex neavei*, ***Culex pipiens*^12b^**^,^**^c^**^,^**^d^**^,^**^e^**, *Culex pseudovishnui*, ***Culex rubinotus*^2^**, *Culex torrentium*, *Culex univittatus*,  *Culiseta morsitans*,  *Mansonia fuscopennata* | Banzi, **Bunyamwera virus^1^**, **Germiston^2^**, Ockelbo, Sindbis, **Usutu^12d^** |
| 10 | ***Aedes canadensis*^14a^**, *Aedes cantator*, *Aedes fulvus*, *Aedes japonicus*, *Aedes mediovittatus*, *Aedes mitchellae*, ***Aedes sollicitans*^14a^**, ***Aedes taeniorhynchus*^14a^**, *Aedes thelcter*, ***Aedes triseriatus*^5^**, ***Aedes trivittatus*^13^**,  *Anopheles grabhamii*, *Anopheles neivai*, *Anopheles neomaculipalpus*, *Anopheles punctipennis*, *Anopheles quadramaculatus*, *Anopheles walkeri*,  ***Coquillettidia perturbans*^14a^**,  *Culex accelerans*, *Culex cedecei*, *Culex corniger*, *Culex dunni*, *Culex gnomatos*, *Culex iolambdis*, *Culex panocossa*, *Culex peccator*, ***Culex pedroi*^14a^**, *Culex sacchettae*, *Culex salinarius*, ***Culex taeniopus*^14a^**^,^**^c^**, *Culex taeniorhynchus*,  *Culiseta melanura*,  *Deinocerites pseudes*,  *Haemagogus* sp*.*,  *Mansonia indubitans*, *Mansonia perturbans*,  *Psorophora cingulata*, *Psorophora columbiae*, *Psorophora confinnis*, *Psorophora discolor*,  *Uranotaenia sapphirina* | Cache Valley Virus, **Eastern Equine Encephalitis^14a^**, Everglades, Guaroa virus, Itaqui Virus, **LaCrosse Encephalitis^5^**, Nepuyo virus, Tacaiuma, Tensaw, **Trivittatus^13^**, **Venezuelan Equine Encephalitis^14c^** |
| 11 | *Aedes albifasciatus*, *Aedes atropalpus*, *Aedes butleri*, *Aedes dorsalis*, *Aedes epaticus*, *Aedes lateralis*, ***Aedes melanimon*^12f^**, *Aedes negromaculis*, *Aedes nigromaculis*, *Aedes sierrensis*,  *Aedes varipalpus*,  *Anopheles brunnipes*, *Anopheles maculipalpis*, *Anopheles plumbeus*, *Anopheles rufipes*, *Anopheles tessellatus*,  *Coquillettidia metallica*, *Coquillettidia microannulata*,  *Culex annulus*, *Culex antennatus*, ***Culex bitaeniorhychus*^12a^**, *Culex coronator*, *Culex decens*, ***Culex declarator*^12b^**, *Culex dorsalis*, *Culex erythrothorax*, *Culex ethiopicus*, *Culex fatigans*, ***Culex fuscocephala*^12a^**, ***Culex gelidus*^12a^**, *Culex guiarti*, ***Culex modestus*^12e^**, ***Culex nigripalpus*^12b^**, *Culex nigripes*, *Culex peus*, *Culex pruina*, ***Culex quinquefasciatus*^12a^**^,^**^b^**^,^**^e^**, *Culex restuans*, *Culex scottii*, *Culex stigmatosoma*, ***Culex tarsalis*^12b^**^,^**^e^**^,^**^f^**, *Culex theileri*, *Culex tritaeniorhychus*, ***Culex vishnui*^12a^**, *Culex weschei*, *Culex whitmorei*,  *Mansonia* sp*.*,  *Mimomyia hispida*, *Mimomyia lacustris*, *Mimomyia splendens*,  *Ochlerotatus geniculatus*,  *Orthopodomyia signifera*,  *Psorophora ciliata*, *Psorophora pallescens*, *Psorophora signipennis*,  *Theobaldia incidens*, *Theobaldia inornata*,  *Wyeomyia vanduzeei* | Banna, California Encephalitis, **Japanese Encephalitis^12a^**, **St. Louis Encephalitis^12b^**, Wanowrie, **West Nile Virus^12e^**, **Western Equine Encephalitis^12f^** |

**Additional file 2: Table S2.** **Associations of vectors and pathogens from cluster analysis** (Figs. 1, 2). Groups identified from analysis of known vectors only are identified in bold, with superscripts corresponding to cluster number.
